# Supplementary material for: A retrospective cohort study of major adverse cardiac events in children affected by Kawasaki disease with coronary artery aneurysms in Thailand
Source: PLoS One. 2022 Jan 27;17(1):e0263060. doi: 10.1371/journal.pone.0263060 (PMC8794099; doi:10.1371/journal.pone.0263060)
Supplement: S1 Table — (DOCX) [file pone.0263060.s002.docx]

**Supplementary data**

**Table S1. Characteristics of 19 patients in the cohort who had major adverse cardiac events (MACE)**

|  | **Time at diagnosis of KD** | **Age at KD (years)** | **Received IVIG (Y/N)** | **Age at MACE report (years)** | **MACE details** |
| --- | --- | --- | --- | --- | --- |
| 1 | 1997 | 2.8 | Yes | 6.9 | Post KD 4 years. Patient had chest pain and ECG showing ST depression in lead V3-V6. CAG showed stenosis at proximal and distal to giant LAD aneurysm and total occlusion of RCA with collateral from OM and LAD. CABG was performed with LIMA to LAD and free graft (RIMA) to PD, PL. |
| 2 | 1997 | 2.2 | Yes | 4.3 | Post KD 2 years. Patient had chest pain and ECG showing ST-T change in V3, V4 with deep Q in I, AVL. CABG was performed showed giant aneurysm at LAD and RCA. Patient underwent CABG; LIMA to LAD and right aneurysmectomy proximal RCA. Seven years later, he had redo CABG; RIMA to mid-RCA. Recently, he was asymptomatic but CTA showed that RIMA was occluded. Adenosine stress perfusion showed fair LV contraction, transmural scar at mid to apical septum and anterior walls, apical inferior and apex, subendocardial scar at basal anteroseptum, mid anterolateral and apical lateral. |
| 3 | 1998 | 1.0 | Yes (Late IVIg Rx) | 6.7 | Patient had giant aneurysms at LMCA, LAD, and RCA. Positive adenosine with stress CMR post KD 5 years; moderate ischemia of septum, inferior and inferolateral wall. CABG was performed LIMA to LAD and RIMA to mid-RCA. |
| 4 | 1999 | 0.3 | Yes | 9.8 | Patient had giant aneurysms at RCA, LAD, and LCx. Dipyridamole stress perfusion scan showed no perfusion deficit initially. At 7 years post KD, patient had chest pain occasionally on exertion, CAG showed increased aneurysm sizes on RCA with mild stenosis distally. Anerysmectomy of mid and distal RCA and end-to-end anastomosis. Patient died in 2019 due to a car accident (passenger). |
| 5 | 2001 | 2.4 | No | 10.1 | Patient had giant aneurysms at RCA and LAD. He was relatively asymptomatic. The MPI showed inferior wall infarction. Low flow in LCx with mild stenosis of proximal RCA. 1^st^ Tc 99m MIBI showed negative perfusion deficit. Post KD 7 years, Tc 99m MIBI showed severe myocardial ischemia + infarction at inferolateral wall apical region. CAG showed near total occlusion of proximal LAD with multiple bridging collateral artery from proximal to mid-LAD with TIMI 3 flows. CABG was performed; LIMA to LAD and SVG to distal LCx. |
| 6 | 2002 | 0.7 | Yes (late IVIg Rx) | 4.5 | Patient had giant aneurysm at RCA with distal stenosis, at LCA extended to LAD and LCx aneurysm with distal obstruction. Stress perfusion CMR showed ischemia of inferior wall and lateral wall suggestive of RCA and LCx distribution at post KD 2 years. CABG was performed RIMA to proximal RCA. |
| 7 | 2003 | 2.7 | Yes (late IVIg Rx) | 3.6 | Patient had giant aneurysms at RCA and LAD. Positive adenosine with stress CMR; severe ischemia at RCA territory and moderate ischemia at LAD territory. CABG was performed LIMA to LAD and SVG to PD. Post CABG 8 years, patient had chest pain and CAG showed CTO at proximal LAD and CTO LIMA graft and patent SVG to RCA. Redo CABG was performed using free graft of RIMA anastomosed to the stump of LIMA and distal RIMA anastomosed to the distal LAD. |
| 8 | 2004 | 5.3 | No | 8.2 | Patient had giant aneurysms at LAD and RCA. CAG showed proximal LAD aneurysm and total occlusion of RCA. Tc99m MIBI showed myocardial ischemia at the anterolateral wall. CABG was performed LIMA to LAD and RIMA to mid RCA. |
| 9 | 2005 | 0.7 | No | 6.7 | Patient had giant aneurysm at RCA and LCx. CAG showed total occlusion of proximal LCx supplied by RCA with severe mid-RCA stenosis. He underwent successful PCI of RCA. |
| 10 | 2006 | 3.0 | Yes (late IVIg Rx) | 8.0 | Patient had giant aneurysm at RCA and small aneurysm at LMCA. He had clinical atypical chest pain post KD 5 years. EST showed ST depression in lead V1, V5. CAG showed total occlusion of RCA with good collaterals flow from LCx. No significant obstruction of LMCA (LAD, LCx). No CABG/PCI was performed. |
| 11 | 2009 | 4.2 | No | 4.3 | Patient had a late diagnosis of KD. No IVIg was given. Post onset KD 3 months, she presented with dysphagia and heart murmur. Giant aneurysms at LAD, LCx and proximal RCA aneurysms with severe MR and fair LV systolic function was revealed on echocardiography. She had positive MIBI; myocardial ischemia at anteroapical wall and inferolateral wall. 1^st^ CABG was performed using SVG to LAD, SVG to OM, RIMA to AM with MV repair. Three years later, she had thrombosis of SVG to LAD graft. 2^nd^ CABG was performed SVG to LAD. |
| 12 | 2009 | 3.7 | Yes (late IVIg Rx) | 4.3 | Patient had giant aneurysms at RCA and LAD. CAG showed multiple giant RCA aneurysms (12 mm, 9 mm, and 7 mm) with partial flow stenosis. Fusiform proximal giant LAD aneurysm (6 mm). MIBI showed mild myocardial ischemia at apex, apical segment of inferior wall and mid-infero lateral wall. He was asymptomatic. Repeat CAG showed no stenosis of RCA and resolved perfusion deficit on MPI. No CABG/PCI was performed. |
| 13 | 2012 | 2.8 | No | 8.5 | Patient had giant aneurysms at RCA, LAD, and LCx. Post KD 5 years, positive for adenosine with stress CMR at inferior + inferoseptal wall. CABG was performed and confirmed stenotic lesion. Patient underwent CABG; LIMA to mid-LAD and RIMA to distal RCA and ligation of distal aneurysm of LCA. |
| 14 | 2012 | 2.1 | Yes | 2.6 | Patient had giant aneurysm at RCA with total occlusion of RCA distal to the aneurysm and collateral artery supplied distal RCA from LAD. Positive adenosine with stress CMR for myocardial ischemia in most of inferior and inferoseptal walls with possible thrombus of prox. RCA. He was asymptomatic. Serial adenosine stress CMR showed negative for ischemia beyond scar. No CABG/PCI was performed. |
| 15 | 2013 | 5.4 | No | 7.4 | Patient had medium-sized aneurysms at LAD and LCx with chronic total occlusion of RCA with collateral supplied from LAD. He was asymptomatic. Positive adenosine with stress CMR with low normal LV function, LVEF of 52% with akinetic mid to apical inferior wall, severe stenosis of proximal to mid-RCA and transmural infarction mid to apical inferior wall, subendocardial infarction at basal inferior wall. No ischemia was found beyond scar. No CABG/PCI was performed. |
| 16 | 2013 | 4.7 | Yes | 10.7 | Patient had giant aneurysm at RCA and LAD. He was asymptomatic. Positive adenosine with stress CMR for myocardial ischemia of mid to apical septal, apical anterior and inferior walls without scar. CAG showed 95% stenosis of proximal LAD and 1^st^ diagonal branch ostial lesion. PCI was performed. |
| 17 | 2014 | 0.3 | Yes (late IVIg Rx) | 2.4 | This boy had giant aneurysm at LMCA and RCA. CTA at 1.8 years post diagnosis showed giant aneurysm with laminated thrombus. He had decreased LV function and HF when he was 2.4 years old. Partial resection aneurysm of LMCA and removed thrombus and CABG was performed using LIMA to LAD and SVG to LCx. |
| 18 | 2015 | 12.5 | No | 12.5 | Patient presented with chest pain and cardiogenic shock from rupture LAD coronary aneurysm with cardiac tamponade. Emergency CABG was performed (SVG to LAD). |
| 19 | 2016 | 0.8 | Yes | 1.5 | Patient had multiple giant aneurysms at RCA. Positive adenosine stress MRI for myocardial ischemia in entire septum; suspicious stenosis of distal RCA. CAG showed less than 25% stenosis mid-RCA. No CABG/PCI therefore was not performed. |

KD=Kawasaki disease; MI=myocardial ischemia; CAAs=coronary artery aneurysms; ECG=electrocardiography; LMCA=left main coronary artery; LAD=left anterior descending artery; RCA=right coronary artery; LIMA=left internal mammary artery; LCx=left circumflex artery; RIMA=right internal mammary; SVG=saphenous vein graft; CAG=coronary angiography; CABG=coronary artery bypass grafting; PCI=percutaneous coronary intervention; CTA=computed-tomography angiography; CMR=cardiovascular magnetic resonance; MIBI scan=myocardial perfusion scan; LVEF=left ventricular ejection fraction.
