# Supplementary material for: A retrospective cohort study of major adverse cardiac events in children affected by Kawasaki disease with coronary artery aneurysms in Thailand
Source: PLoS One. 2022 Jan 27;17(1):e0263060. doi: 10.1371/journal.pone.0263060 (PMC8794099; doi:10.1371/journal.pone.0263060)
Supplement: S1 File — (ZIP) [file pone.0263060.s003.zip › original IRB and research proposal 2018/Translated Document No. 2 IRB Submission Form MACE KD_JUN2018.docx]

Document no.2

**IRB Submission Form**

**Faculty of Medicine Siriraj Hospital**

**Protocol identification and Investigator**

1. **Project title (Thai)** ภาวะแทรกซ้อนทางระบบหัวใจและหลอดเลือดในผู้ป่วยโรคคาวาซากิที่มีหลอดเลือดหัวใจโป่งพอง.....

**(English)** Cardiac events in Kawasaki disease with coronary aneurysms revisited.........

1. **Name of Principal Investigator (Thai)** รศ.พญ. ชดชนก วิจารสรณ์...............................

**(English)**.. Chodchanok Vijarnsorn MD................

Instructor Academic title.....Associate professor....................................

Resident  Fellow

Student Degree  Bachelor  Master’s  PhD

Other personnel: Position.....................................................

Educational degree.....MD....................................................................

Faculty of Medicine Siriraj Hospital, Mahidol University

Telephone (which can be reached in and after office hours) .081-3447015..........

E-mail address: cvijarnsorn@yahoo,com.............

(Please also provide your curriculum vitae)

- 1. **Responsible research work**

Number of research projects currently under responsibility………3……………projects

Expected number of participants/volunteers under responsibility totaling…………3…………. persons.

**Experiences in ethical issues in research involving human subjects**

Experiences in research ethics training in (year) ……2018…………..… (attached the certificate)

Experiences in Good clinical practice training in (year) ………… (attached the certificate)

(Researcher should attend the training once every three years.)

- 1. Researcher’s conflict of interest with organization sponsoring the research/ research drugs/research devices (such as having share/or family members have shares in the sponsoring company, being a consultant, obtaining honoraria, travel reimbursement, other financial support from the funding source)

No  Yes, (more than 300,000 baht/ yr. please attached document no.14)

1. **All co-researchers**
   1. **Name of co-researcher (Thai)** พญ. กนกวลี สันติมหกุลเลิศ.................................

**(English)** Kanokvalee Santimahakullert MD.

Instructor Academic title ............................................................................................................................

Resident  Fellow

Student Degree  Bachelor  Master’s  PhD

Other personnel: Position .....................................................

Educational degree ...MD...................................................................

Faculty of Medicine Siriraj Hospital, Mahidol University

Telephone (which can be reached in and after office hours).086-7874414.................

E-mail address: ... kanokvalee@hotmail.com....................................................

(Please also provide your curriculum vitae)

- **Experiences in ethical issues in research involving human subjects**

Experiences in research ethics training in (year) …2017…………..…… (attached the certificate)

Experiences in Good clinical practice training in (year) ………… (attached the certificate)

*Please provide the document with reasons to IRB committee in case of no certificate in ethics training.*

- **Co-researcher’s conflict of interest with organization sponsoring the research/ research drugs/research devices** (such as having shares/or family members have shares in the sponsoring company, being a consultant, obtaining honoraria, travel reimbursement, other financial support from the funding source)

No  Yes, (more than 300,000 baht/ yr. please attached document no.14)

- 1. **Name of co-researcher (Thai)** นศพ. สัภยา ครองศรัทธา

**(English)** Mr. Sappaya Krongsrattha

Instructor Academic title ............................................................................................................................

Resident  Fellow

Student Degree  Bachelor  Master’s  PhD

Other personnel: Position Medical student, Faculty of Medicine Siriraj Hospital, Mahidol University...........

Educational degree ......................................................................

Faculty of Medicine Siriraj Hospital, Mahidol University

Telephone (which can be reached in and after office hours). 084-3776856.................

E-mail address: …sappayakhrongsrattha@gmail.com.....................................................

(Please also provide your curriculum vitae)

- **Experiences in ethical issues in research involving human subjects**

Experiences in research ethics training in (year) …2017…………..…… (attached the certificate)

Experiences in Good clinical practice training in (year) ………… (attached the certificate)

*Please provide the document with reasons to IRB committee in case of no certificate in ethics training.*

- **Co-researcher’s conflict of interest with organization sponsoring the research/ research drugs/research devices** (such as having shares/or family members have shares in the sponsoring company, being a consultant, obtaining honoraria, travel reimbursement, other financial support from the funding source)

No  Yes, (more than 300,000 baht/ yr. please attached document no.14)

- 1. **Name of co-researcher (Thai)** ผศ. นพ. ยุทธพงศ์ วงศ์สวัสดิวัฒน์...............................

**(English)** Yuttapong Wongswadiwat MD

Instructor Academic title ...Assistant professor...............................

Resident  Fellow

Student Degree  Bachelor  Master’s  PhD

Other personnel: Position .....................................................

Educational degree ...MD...................................................................

Faculty of Medicine, Srinagarind Hospital, Khon Kaen University, Khon Kaen

Telephone (which can be reached in and after office hours) 084-6018199.................

E-mail address: ... nung100@yahoo.com....................................................

(Please also provide your curriculum vitae)

- **Experiences in ethical issues in research involving human subjects**

Experiences in research ethics training in (year) …2017…………..…… (attached the certificate)

Experiences in Good clinical practice training in (year) ………… (attached the certificate)

*Please provide the document with reasons to IRB committee in case of no certificate in ethics training.*

- **Co-researcher’s conflict of interest with organization sponsoring the research/ research drugs/research devices** (such as having shares/or family members have shares in the sponsoring company, being a consultant, obtaining honoraria, travel reimbursement, other financial support from the funding source)

No  Yes, (more than 300,000 baht/ yr. please attached document no.14)

- 1. **………………………………………………………………………………………..…………………….**

1. **Research funding**

No  Applying for funding (please specify the funding source)………………..………………

Research fund was granted*

Government (please specify) …………………...………….……..….…….……..….……..……..………...

Private (please specify) ………………………………...……………………………..……………….……….....

NGO (please specify) ………………………………….…………..………...……………….………….……….…

Other (please specify) ………………………………………......….…………………….…………………………

Address of funding source ..............................................................................................................................

Name of coordinator of the funding source..................................................................................................

Telephone (which can be reached in and after office hours)...................................................................

E-mail address: .....................................................................................................................................................

1. **Research site**

Single center, please specify...............................................................................................................................

Multiple centers

only in Thailand

MOU in Mahidol University  No  Yes

MOU _Central Research Ethics Committee (CREC)  No  Yes

(please specify every institute that takes part in the research project, number of participants/volunteers and the result of IRB consideration in each institute) ....................................................................................................

collaboration with overseas

(please specify the country and every institute in Thailand that take part in the research project, with number of participants/volunteers and the result of IRB consideration in each institute in Thailand)

.....................................................................................................................................................................................................

1. **Duration of research project**

For the whole project …………….…2…………....………Years........................Months

Duration of data collection ……………1.………………Years........................Months

(Researcher can start collecting data only after obtaining the SIRB approval.)

1. **This research is a part of education: for degree, diploma or independent study**

No

Yes please specify.

Physician who has to work in the provincial hospital for 3 years after graduation/resident

Fellow

Bachelor  Master  PhD

Obtained the approval from the program committee (Thesis) or supervisor

**date** obtained ..28 NOV 2017...................................  not obtained

1. **Summary of proposal**

**Submit with the full proposal (if any)**

- 1. **Background/Rationale**

(Please explain the important content; the content should comply with the research proposal if you have attached it.)

Kawasaki disease (KD) is an acute febrile vasculitis of unknown etiology that commonly occurs in young children. It is recognized to relate with coronary artery lesion (CAL) or coronary artery aneurysms (CAAs) in 15% to 25% if left untreated ^(^[^1-3^](#_ENREF_1)^)^. Timely initiation with intravenous immunoglobulin (IVIG) has reduced the incidence of coronary artery aneurysms defined from absolute luminal dimensions to 4-10% ^(^[^1^](#_ENREF_1)^,^ [^3-5^](#_ENREF_3)^)^. Long-term complication is determined by the initial and progression of coronary artery involvement on the current follow up. Approximately one half of patients, CAAs appear to resolve within 1 to 2 years. On the other hand, some patients had persistent aneurysms which subsequently lead to thrombosis and stenotic lesions that result in myocardial ischemia and infarction ^(^[^2^](#_ENREF_2)^)^. Aggressive management such as thromboprophylaxis and revascularization intervention might be required for complicated and selected patients ^(^[^4^](#_ENREF_4)^,^ [^6^](#_ENREF_6)^,^ [^7^](#_ENREF_7)^)^.

Since American Heart Association (AHA) published guidelines for the diagnosis, treatment, and long term management of KD in 2004 and recently in 2017, criteria for diagnosis of typical and suspicious KD have been widely recognized ^(^[^3^](#_ENREF_3)^,^ [^6^](#_ENREF_6)^)^. An algorithm ensuring captures of incomplete KD in purpose to manage the patients at risk in the effective window of therapy. This has been improved management of the acute illness in addition to acknowledgment of the care that is needed in the long term especially in adults with a previous history of KD and coronary artery aneurysms ^(^[^8^](#_ENREF_8)^)^. The latest guideline stratified patients into 5 risk levels according to their relative risk of myocardial ischemia and infarction and indicated subset each risk level using current status of coronary artery ^(^[^4^](#_ENREF_4)^,^ [^6^](#_ENREF_6)^,^ [^9^](#_ENREF_9)^)^. Serial echocardiography is recommended for patients without CAAs or with transient coronary artery dilatations normalizing within the first 6 to 8 weeks after the acute presentation of the disease ^(^[^3^](#_ENREF_3)^,^ [^4^](#_ENREF_4)^,^ [^6^](#_ENREF_6)^)^. For patients with persistent CAAs, serial myocardial stress tests are recommended in addition to regular echocardiography (risk levels III–V) ^(^[^4^](#_ENREF_4)^)^.

A large Japanese nationwide survey between 1999 and 2010 ^(^[^10^](#_ENREF_10)^)^ identified 209 patients with giant aneurysms with the 10-year survival rate of 94.3% and the total cardiac event-free rate of 0.68. Mortality rate was 5.7% (12/209) and 83.3% occurred within 1 year ^(^[^10^](#_ENREF_10)^)^. A large 2‐center retrospective study of 500 CAAs in 2,860 KD patients in US between 1979 and 2014 ^(^[^11^](#_ENREF_11)^)^reported that 75% had CAA regression within 2 years of KD episode and major adverse cardiac events (MACE) occurred in 24 patients (3 deaths, 1 orthotopic heart transplant (OHT), 6 coronary bypass graft surgery, 2 percutaneous coronary intervention, 12 findings of coronary occlusion). Lack of IVIG treatment and larger CAA size at diagnosis were associated with MACE in multivariable analysis ^(^[^11^](#_ENREF_11)^)^. In Thailand, incidence of KD was from 2.14 to 3.43 cases per 100,000 children aged 0-5 years ^(^[^12^](#_ENREF_12)^)^. Multicenter study of KD patients in 1998 – 2002 reported that 15.6% of 435 patients were resistant cases^(^[^12^](#_ENREF_12)^)^. Prevalence of incomplete KD was reported of 29% (61/208) in a single center study from Northern Thailand^(^[^13^](#_ENREF_13)^)^. These represented increased risk of CAA in Thai population. Base on the latest KD guideline, definition of CAA including dimensional z- score and risk stratification has been introduced. A long‐term outcome of CAA after treatment with IVIG however is lack in Thai population. We therefore conduct this surveillance to revisit a natural history of CAA in a cohort of KD patients and to identify factors associated with MACE in Thai population using KD database of 2 large cardiac centers (Siriraj Hospital and Khonkhan University hospital).

**References**

1. Durongpisitkul K, Gururaj VJ, Park JM, Martin CF. The prevention of coronary artery aneurysm in Kawasaki disease: a meta-analysis on the efficacy of aspirin and immunoglobulin treatment. Pediatrics. 1995;96:1057-61.

2. Kato H, Sugimura T, Akagi T, Sato N, Hashino K, Maeno Y, et al. Long-term consequences of Kawasaki disease. A 10- to 21-year follow-up study of 594 patients. Circulation. 1996;94:1379-85.

3. Newburger JW, Takahashi M, Gerber MA, Gewitz MH, Tani LY, Burns JC, et al. Diagnosis, treatment, and long-term management of Kawasaki disease: a statement for health professionals from the Committee on Rheumatic Fever, Endocarditis and Kawasaki Disease, Council on Cardiovascular Disease in the Young, American Heart Association. Circulation. 2004;110:2747-71.

4. McCrindle BW, Rowley AH, Newburger JW, Burns JC, Bolger AF, Gewitz M, et al. Diagnosis, Treatment, and Long-Term Management of Kawasaki Disease: A Scientific Statement for Health Professionals From the American Heart Association. Circulation. 2017;135:e927-e99.

5. Shah V, Christov G, Mukasa T, Brogan KS, Wade A, Eleftheriou D, et al. Cardiovascular status after Kawasaki disease in the UK. Heart. 2015;101:1646-55.

6. Newburger JW, McCrindle BW, Rowley AH, Burns JC, Bolger AF, Gewitz M, et al. Kawasaki disease: State of the art

Diagnosis, Treatment, and Long-Term Management of Kawasaki Disease: A Scientific Statement for Health Professionals From the American Heart Association. Congenit Heart Dis. 2017;12:633-5.

7. Chanthong P, Sriyoschati S, Durongpisitkul K, Soongswang J, Laohaprasitiporn D, Nana A. Coronary artery bypass graft in Kawasaki disease patients: Siriraj experience. J Med Assoc Thai. 2005;88 Suppl 8:S197-202.

8. Freeman AF, Shulman ST. Kawasaki disease: summary of the American Heart Association guidelines. Am Fam Physician. 2006;74:1141-8.

9. JSC RCo. Guidelines for medical treatment of acute Kawasaki disease: report of the Research Committee of the Japanese Society of Pediatric Cardiology and Cardiac Surgery (2012 revised version) Guidelines for diagnosis and management of cardiovascular sequelae in Kawasaki disease (JCS 2013). Digest version. Pediatr Int. 2014;56:135-58.

10. Fukazawa R, Kobayashi T, Mikami M, Saji T, Hamaoka K, Kato H, et al. Nationwide Survey of Patients With Giant Coronary Aneurysm Secondary to Kawasaki Disease 1999-2010 in Japan. Circ J. 2017;82:239-46.

11. Friedman KG, Gauvreau K, Hamaoka-Okamoto A, Tang A, Berry E, Tremoulet AH, et al. Coronary Artery Aneurysms in Kawasaki Disease: Risk Factors for Progressive Disease and Adverse Cardiac Events in the US Population. J Am Heart Assoc. 2016;5.

12. Durongpisitkul K, Sangtawesin C, Khongphatthanayopthin A, Panamonta M, Sopontammarak S, Sittiwangkul R, et al. Epidemiologic study of Kawasaki disease and cases resistant to IVIG therapy in Thailand. Asian Pac J Allergy Immunol. 2006;24:27-32.

13. Sittiwangkul R, Pongprot Y, Silvilairat S, Makonkaewkeyoon K. Clinical spectrum of incomplete Kawasaki disease in Thailand. Paediatr Int Child Health. 2013;33:176-80.

...................................................................................................................................................................................

...................................................................................................................................................................................

- 1. **Objectives**

**-** To report major adverse cardiac events (MACE) rates in KD patients with CAAs using 2017 KD guideline definition.

**-** To assess predictors of major adverse cardiac events (MACE) in Thai population with CAAs following KD.

...................................................................................................................................................................................

- 1. **Research category** (can choose more than one items)

Experimental biomedical / clinical research: please specify

Drug trial phase.....................please specify drug name..................................................................

Registered drug (please attach drug registration or drug leaflet)

Investigational (new) drug

Drug storage ……………………………………..registered drug storage  yes  no

(Please registered drug storage to the Siriraj Hospital Pharmacy Department, register form is on website of SIRB)

Medical device trial, please specify device name .................................................................................

Registered device (please attach device registration or device leaflet)

Investigational (new) device

Place to store study drugs/vaccine…………………………………………. Register to Pharmacy

Department  Yes  No If No, please register storage place with Pharmacy Department by using registration form on SIRB website.

Vaccine trial phase.................... please specify name/code of vaccine.................................................

Registered vaccine (please attach vaccine registration or vaccine leaflet)

Investigational (new) vaccine

Experimental procedure / intervention, please specify...........................................................................

High Risk  Minimal Risk

Bioequivalence

*In vitro* / laboratory-based study

Research using repository of biological products (cells, blood, tissues, fluids, etc.)

*Specify kind/quantity/number of products use ......................................................................................... (Attach the permission to use repository of biological products from Head of Department / Division)

Others .....................................................................................................................................................

Observation clinical research

Prospective (cohort) study

Case series

Retrospective (chart) review

(Attach the letter of permission to use the medical records from Head of Department/Division with the submission form)

Epidemiology Research

Surveillance

Monitoring

Others (please specify) ...............................................................................................................

Social / Behavioral research

Questionnaire-based research

Others (please specify)……………………………………………….. ...........................................

- 1. **Research design**

Randomized-controlled trial

Quasi-experimental study (manipulation and control only, without randomization)

Pre-experimental study (manipulation only, without control and randomization)

Prospective cohort study

Descriptive study

Cross-sectional study

Pilot study

Others(please specify).....................................................................................................................

- 1. **Research subjects**

**Sample size calculation,** please specify the background of sample size in each group. If the fixed formula has been used, please show the formula for calculation and indicate the variables used in the formula with references

**Base on previous publication** ^(11)^, prevalence of CAAs in children with KD was reported of 17%.


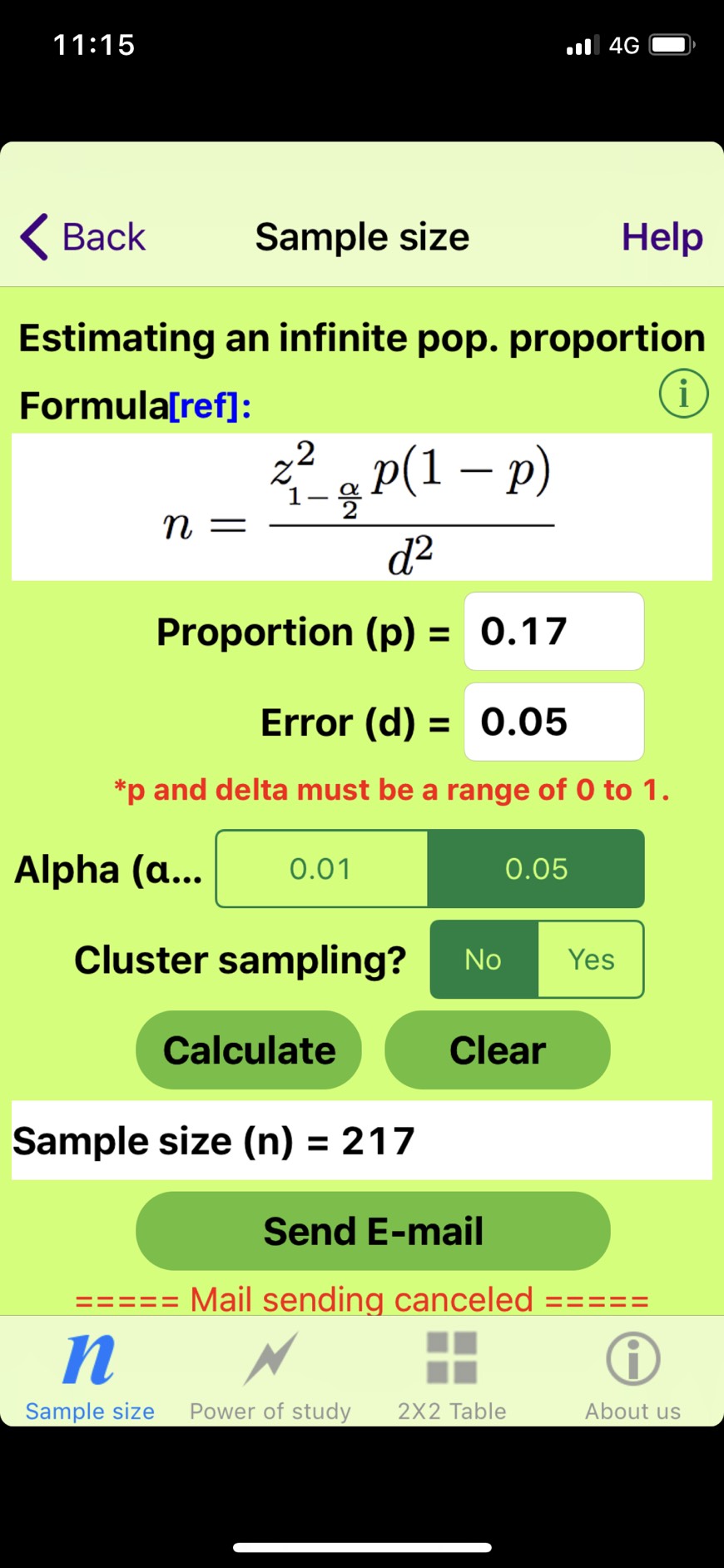


...............................................................................................................................................................................

**Inclusion criteria**

1. Patients who previously diagnosed of Kawasaki disease complicated with coronary artery aneurysms in two centers (Siriraj Hospital and Khonkhan University Hospital) between 2007 and 2017

2. Patients who diagnosed of Kawasaki disease coronary artery aneurysm and clinically follow up at least a year

**Exclusion criteria**

Patients with incomplete initial echocardiographic data.

Patients with co-morbidity of other cardiovascular diseases such as congenital heart defect.......

**Withdrawal or termination criteria**

N/A due to a retrospective trial.......................

**Subject allocation**

N/A due to a retrospective trial.........................

- 1. **Research Process**

Please specify research procedures, research devices, steps of research process, things that participants/volunteers must do or be treated (such as number of blood drawn, amount of blood drawn, number of appointments, time consuming for participation in the study). If the research proposal is attached, the wordings in Thai and English versions must be the same, including the reference pages in the relevant proposal.

Data Collection**:** (please see CRF)

Pertinent data will incorporate the following parameters:

1. Demographic information: age, gender, date of birth, initial diagnosis, initial weight, height, BSA

2. Clinical data: date of diagnosis, presentation at initial diagnosis, initial laboratory including; ESR, CRP, CBC, ALT, albumin, concomittent infection, presence of heart failure, cardiogenic shock,

3. Echocardiographic assessment:

Initial echocardiography: date of echocardiography, LVEF, presence of LV systolic function impairment, initial coronary involvement,

Echo Dx CAAs: date of diagnosis of CAAs, LVEF, presence of LV systolic function impairment, initial coronary involvement,

Echo at 8 weeks post diagnosis: progression

Echo at 1 year post KD: progression

4. Treatment: date of IVIg, receiving 2^nd^ dosage IVIg, date of repeat IVIg, adjunctive anti-inflammatory medications

5. Other tests:

- CAG findings, coronary stenosis present or not

- Stress MPI assessment: type, findings

6. Clinical outcomes in 1 year post KD including major adverse cardiac events: date of 1 year follow up, NYHA, coronary intervention, cardiac death, medications, date of surgical procedure, intervention procedure, date of intervention procedure, current cardiac problem; symptoms; chest pain, syncope, arrhythmia

7. Recent clinical outcomes in 1 year post KD including major adverse cardiac events: date of 1 year follow up, NYHA, coronary intervention, cardiac death, medications, date of surgical procedure, intervention procedure, date of intervention procedure, current cardiac problem; symptoms; chest pain, syncope, arrhythmia, recent risk level, nutritional status, recent lipid profile, recent blood pressure, smoking, co-morbid.

Definition of CAA and MACE (also presented in the last page of CRF)

| Items | Definition |
| --- | --- |
| Late diagnosis and treated KD | KD which initiated treatment at >= 10 days of onset of fever  *Ref: AHA KD guideline 2017* |
| Coronary involvement and CAA criteria (echo, CAG) | Using Z-Score Classification  1. No involvement: Always <2  2. Dilation only: 2 to <2.5; or if initially <2, a decrease in Z score during follow-up ≥1  3. Small aneurysm: 2.5 to <5  4. Medium aneurysm: 5 to <10, and absolute dimension <8 mm  5. Large or giant aneurysm: > 10, or absolute dimension >8 mm  Remarks: One potential limitation of this study is that regression formulas for the LAD were used to derive Z scores for the left circumflex branch (normal values for the circumflex are not available with the Z-score system that was used).  *Ref: AHA KD guideline 2017* |
| Risk level of KD (based on 2017 KD guideline) | 1: No involvement,  2: Dilation only  3.1: Small aneurysm, current or persistent  3.2: Small aneurysm, regressed to normal or dilation only  4.1: Medium aneurysm, current or persistent  4.2: Medium aneurysm, regressed to small aneurysm  4.3: Medium aneurysm, regressed to normal or dilation only  5.1: Large and giant aneurysm, current or persistent  5.2: Large or giant aneurysm, regressed to medium aneurysm  5.3: Large or giant aneurysm, regressed to small aneurysm  5.4: Large or giant aneurysm,regressed to normal or dilation only  *Ref: AHA KD guideline 2017* |
| MACE (major adverse cardiac events | All-cause mortality or re-hospitalization for a cardiovascular-related illness. Cardiovascular-related illnesses included heart failure, reinfarction (nonfatal), recurrence of angina pectoris and repeat PCI or CABG  *Ref :*  *Califf RM, Bengtson JR. Cardiogenic shock. N Engl J Med. 1994;330:1724–30.*  *Sai IT, Wang CP, Lu YC, et al. BMC Cardiovasc Disord. 2017 Jan 4;17(1):1.* |

................................................................................................................................................................................................................

................................................................................................................................................................................................................

- 1. **Data collection process**

Please provide case record form, and/or questionnaire, and/or interview question, and/or telephone script to obtain consideration (if any).

The CRF is attached.

**Data collection must be started after obtaining the SIRB approval**

The case record form must not indicate name, Hospital Number (HN) or any identifications that link to individual subjects,(using the code instead).

….............................................................................................................................................................................................................

- 1. **Outcome measurement / data analysis,** including statistics used in the research

Patients’ baseline characteristics will be summarized using descriptive statistic presenting in percentage, mean and standard deviation. CMR results will be correlated to epicardial vessels patency. The association of cardiac events and perfusion defect will be determined by risk analysis.

...............................................................................................................................................................................................................

- 1. **Evidence, data or references** (writing references should be complied with the international standard)

1. Durongpisitkul K, Gururaj VJ, Park JM, Martin CF. The prevention of coronary artery aneurysm in Kawasaki disease: a meta-analysis on the efficacy of aspirin and immunoglobulin treatment. Pediatrics. 1995;96:1057-61.

2. Kato H, Sugimura T, Akagi T, Sato N, Hashino K, Maeno Y, et al. Long-term consequences of Kawasaki disease. A 10- to 21-year follow-up study of 594 patients. Circulation. 1996;94:1379-85.

3. Newburger JW, Takahashi M, Gerber MA, Gewitz MH, Tani LY, Burns JC, et al. Diagnosis, treatment, and long-term management of Kawasaki disease: a statement for health professionals from the Committee on Rheumatic Fever, Endocarditis and Kawasaki Disease, Council on Cardiovascular Disease in the Young, American Heart Association. Circulation. 2004;110:2747-71.

4. McCrindle BW, Rowley AH, Newburger JW, Burns JC, Bolger AF, Gewitz M, et al. Diagnosis, Treatment, and Long-Term Management of Kawasaki Disease: A Scientific Statement for Health Professionals From the American Heart Association. Circulation. 2017;135:e927-e99.

5. Shah V, Christov G, Mukasa T, Brogan KS, Wade A, Eleftheriou D, et al. Cardiovascular status after Kawasaki disease in the UK. Heart. 2015;101:1646-55.

6. Newburger JW, McCrindle BW, Rowley AH, Burns JC, Bolger AF, Gewitz M, et al. Kawasaki disease: State of the art

Diagnosis, Treatment, and Long-Term Management of Kawasaki Disease: A Scientific Statement for Health Professionals From the American Heart Association. Congenit Heart Dis. 2017;12:633-5.

7. Chanthong P, Sriyoschati S, Durongpisitkul K, Soongswang J, Laohaprasitiporn D, Nana A. Coronary artery bypass graft in Kawasaki disease patients: Siriraj experience. J Med Assoc Thai. 2005;88 Suppl 8:S197-202.

8. Freeman AF, Shulman ST. Kawasaki disease: summary of the American Heart Association guidelines. Am Fam Physician. 2006;74:1141-8.

9. JSC RCo. Guidelines for medical treatment of acute Kawasaki disease: report of the Research Committee of the Japanese Society of Pediatric Cardiology and Cardiac Surgery (2012 revised version) Guidelines for diagnosis and management of cardiovascular sequelae in Kawasaki disease (JCS 2013). Digest version. Pediatr Int. 2014;56:135-58.

10. Fukazawa R, Kobayashi T, Mikami M, Saji T, Hamaoka K, Kato H, et al. Nationwide Survey of Patients With Giant Coronary Aneurysm Secondary to Kawasaki Disease 1999-2010 in Japan. Circ J. 2017;82:239-46.

11. Friedman KG, Gauvreau K, Hamaoka-Okamoto A, Tang A, Berry E, Tremoulet AH, et al. Coronary Artery Aneurysms in Kawasaki Disease: Risk Factors for Progressive Disease and Adverse Cardiac Events in the US Population. J Am Heart Assoc. 2016;5.

12. Durongpisitkul K, Sangtawesin C, Khongphatthanayopthin A, Panamonta M, Sopontammarak S, Sittiwangkul R, et al. Epidemiologic study of Kawasaki disease and cases resistant to IVIG therapy in Thailand. Asian Pac J Allergy Immunol. 2006;24:27-32.

13. Sittiwangkul R, Pongprot Y, Silvilairat S, Makonkaewkeyoon K. Clinical spectrum of incomplete Kawasaki disease in Thailand. Paediatr Int Child Health. 2013;33:176-80.

...............................................................................................................................................................................................................

**Ethical Consideration**

1. **Characteristics of participants/volunteers**

Healthy volunteers

Patients excluding vulnerable subjects

Others such as Retrospective chart review

Vulnerable subjects*, please specify the subject who cannot make decision by himself or

children under 18 years **

disabled

emergency patient or ICU patient, palliative care patient

chronic patient who must be in care of doctor or care taker

pregnant woman  students  prisoners

armed force  person in the foster home  illiterate person

others (please specify) …………........................................................................................................................

Describe additional safeguards to protect the rights and welfare of vulnerable subject .………………………………………………………………………….……………………………………………………………………………………………………………………

* If the participants/volunteers are the vulnerable subjects and the researcher needs the consent from the legal representative, to whom he expects to obtain the consent.

Please specify ………….............................................................................................................................................................................

** In case of children ages 7-12 years, the assent may be obtained directly from the children, in addition to the consent from guardian or legal representative.

** In case of children ages over 12 – under 18 years, the assent must be obtained directly from the children, in addition to the consent from guardian or legal representative (except in some cases such as mental retardation)

1. **The usage of medical records/specimens of the participants/volunteers**
   1. Having permission for using repository of medical products from authorized person

No  Yes  not related

- 1. Having permission for using participant/volunteer specimens for future used

No  Yes  not related

- 1. Having specimens sending out of the institute

No  Yes  not related

Having specimens sending into the institute

No  Yes  not related

(The Material Transfer Agreement must be performed in both cases of no 10.3 and a copy must be provided to the SIRB committee prior to obtaining the SIRB certificate of approval.)

1. **Recruitment process**

**11.1 Research site:** Please specify where and how potential subjects will be approached for participation in this study: Retrospective chart review in Faculty of medicine Siriraj hospital, Mahidol University, Bangkok and Faculty of Medicine, Srinagarind Hospital, Khon Kaen University, Khon Kaen, Thailand

**11.2 Process**

**11.2.1** A person who invites the volunteers to take part in the research

Principal investigator  Co-investigator  Research assistant (such as research nurse, student)

Physician in charge of the patient  Others: N/A due to a retrospective chart review............................

* The principal investigator who himself provides treatment to the patient should not invite the patient to take part in the research directly because it might cause undue influence. To minimize the possibility of coercion or undue influence, the persuader and basic information provider must not be directly an influential person to the participants/volunteers.

**11.2.2** Describe in detail the invitation process for participants to take part in the research such as the approach to participants/volunteers, data access, including the tools used in the invitation and media use (if any);* attach the advertisement of subject recruitment and telephone script for consideration

- N/A due to a retrospective chart review…………………………………………………………………..

*Media use such as the advertisement of subject recruitment and telephone invitation must contain the wordings showing that the participant/volunteer’s participation is voluntary without undue influence. (The advertisement must contain the approval stamp before advertising.)

1. **Informed consent process**

Not related owing to being the retrospective chart review

A waiver of consent form (attach Document No. 1e)

Apply for the informed consent, please specify the following information.

🞍The person who will provide consent or permission………….………………………………………………………….………………

🞍Any waiting period between informing the prospective participant and obtaining consent………………..………

Participant have opportunity to consider before obtain the consent ……………………….……..………………………….…

🞍The language understood by the prospective participant or the legally authorized representative………..…

🞍The language used by those obtaining consent ……………………………………………….…………

🞍How to keep subject’s privacy and confidentiality,  Where ...........................................

- 1. **Relevant documents** (you can choose every relevant item)
- N/A due to a retrospective chart review............................

Participant information sheet (Document No. 3a) and  Informed consent form (Document No. 3b)

Participant information sheet and assent form for children ages 7-12 years (Document No. 4.1) attach with the participant information sheet and consent form from guardian/legal representative (Document No. 3a and 3b by changing pronoun appropriately)

Participant information sheet and assent form for children ages over 12- under 18 years (Document No. 4.2) attach with the participant information sheet and consent form from guardian/legal representative (Document No. 3a and 3b by changing pronoun appropriately)

- 1. **Process**: Describe in detail about the consent form process from the volunteer and/or legal representative

N/A due to a retrospective chart review............................

**12.2.1** A person who asks for the consent from the volunteers for taking part in the research (you can choose every relevant item).

Principal investigator  Co-investigator  Research assistant (such as research nurse, student)

Physician in charge of the patient  Others, N/A due to a retrospective chart review............................

**12.2.2** Describe in detail the informed consent form process from the participant/volunteer’s or legal representative… N/A due to a retrospective chart review............................

*In case of obtained informed consent form legally representative. Research team must reobtain informed consent again (reconsent) from research participant whenever his/her ability is regained.

1. **Benefits expected to gain from research**
   1. Benefit to individual participant/volunteer: This is a retrospective observational study, the investigator will review and know the progression of disease.
   2. Benefit to profession as a whole: The findings from the study will enhance physicians in caring the patients who had KD with CAAs.

**13.3** Benefit to social welfare: The knowledge from this study such as risk of MACE will add the caution in caring the patients who had KD with CAAs.

**13.4** Others …………………………………….………………………….…………………………………………..

1. **The impact which may occur to participant/volunteer and compensation**
   1. Describe whether there are the physical, mental, social and economy impacts especially the risk, or not. (Are there any research identical to the proposed proposal and any adverse events occurred? Please explain in details the opportunity that the adverse event may occur from the information and reviewer assessment)

including the inconvenience and wasting time

- There is no or minimal risk to the participants due to a retrospective chart review..........
  1. Regulation for protection and correction as well as additional safeguards to protect the participants rights and welfare prepared by the researcher if the adverse event occurs
- N/A due to a retrospective chart review
  1. The person who is responsible for the expenses/compensation on the correction or treatment incurred by the adverse event. If there is an insurance policy.*(If the researcher is a faculty employee and does not obtain funding from the private agency, he can indicate Siriraj Hospital as responsible organization in case of adverse event occurs)*
- Chodchanok Vijarnsorn MD. Tel 081-3447015*.*..........................................................................................................
  1. Expenses that the participant/volunteer must be responsible and the compensation he will obtain

- N/A due to a retrospective chart review....................................................

- 1. Name of responsible person or physician and telephone number that can be reached at all time in case that the adverse event occurs from the research

- Chodchanok Vijarnsorn MD. Tel 081-3447015................................................................................................................

- 1. How does the researcher inform the physician in charge or other physicians who provide treatment to the participant/volunteer about his taking part in the research if it is the clinical trial or the research used the diagnostic results such as laboratory, pathological and radiological results?
- The research proposal was presented to all primary cardiologists in the center in the division meeting on 19 OCT 2017 and the research was permitted to perform.

………………...............................................................................................................................................................................................

Remarks: in study with intervention to the participants, researcher should put the study code, investigator name, and telephone number in patient's medical records.

- 1. The research that is monitored by the Study Monitoring and, Data Safety Monitoring Board (DSMB)

Yes, please specify.......................................................

No  Not related

- 1. Other alternative treatments

Yes, please specify.......................................................

No  Not related

- 1. Is there any plan of interim analysis for the risk of the whole project?

Yes, please specify.......................................................

No  Not related

1. **Does the research involve or impact on the religion, belief, tradition, culture or good reputation of the institute, local or country that the research is conducting?**

Related to, please specify the protection and the way to reduce such impact......................................

Not related

1. **The method that will ensure the confidentiality or privacy of participant/volunteer** (Tick ☑ to every relevant item)
   1. a Will the consent process take place with the subject in a private area or room?

yes  no (if not, explain: N/A due to retrospective chart review________)

b Will the study procedures take place in a private area or room?

yes  no (if not, explain: N/A due to retrospective chart review________)

**16.2** Personal case record

No personal case record of participant/volunteer

Having personal case record of participant/volunteer (must answer item 16.3)

Please use the code instead of name and personal data of participant/volunteer with no specification of date, month and year of birth, the initial letter of the first name and the last name

Electronic file  Photos/still photos  Video/moving images

Audio tapes  Others, please specify ........................................................................

**16.3** If the personal information has been used as mentioned above, please specify the persons who can access the information, duration of data collection, and the method of data destruction after the completion of research.

Record in the personal computer with protection code

Keep document / CD / files in the locked cabinet and only the researcher has a key to open or close

Destroy the documents / CD / files after the research is complete

Forward CD of patient’s history back to the Medical Statistics Division after the research is complete

Keep document / CD / files for……….years after the research is complete

Others, please specify ....................................................................................................................................

Please specify the persons who can access the information ………………………………………………..……….

Principal investigator is responsible for maintaining the confidentially of the participant/volunteer informations and it must be stated in the participant information sheet.

**Researcher’s statement**

The principal and all co-investigators, whose names appeared in Item number 2 and 3, must review the processes in ethical research conducts at Faculty of Medicine Siriraj Hospital vice infra, and confirm your acceptance of the terms and conditions by checking ✓in front of each item. The document must be signed and dated at the bottom prior to submission.

..✓.. 1) All investigators will conduct the research based on the Researcher’s Code of Ethics, the ethical criteria of human research studies, and the appropriate Faculty principles, in intention to maintain prestige and honor within the researchers;

..✓.. 2) All investigators will conduct the research according to what stated in the research approved by the SIRB committee, and will use the IRB approved documents to obtain the informed consent from the participant/volunteer correctly with the respect to the dignity, right and welfare of the participant/volunteer significantly;

..✓.. 3) All investigators have good knowledge and understanding in every step of the proposed research process, and are capable to solve the problems or adverse event which may occur during conducting the research in order to ensure the safety and welfare of participant/volunteer;

..✓.. 4) All investigators will not initiate the research study before the official approval of the Siriraj Institutional Review Board;

..✓.. 5) All investigators understand that we will have access for data entry and management that is confidential. All personally identifying records developed or acquired in the course of the research study will be kept confidential and will not be disclosed by any person in possession of the record, nor will these records be discoverable by persons not involved in the research project;

..✓.. 6) All investigators will report the serious/unexpected adverse event during conducting the research according to the SIRB regulations within the course, and will provide assistance to solve the problems at the best of our ability;

..✓.. 7) All investigators will inform the SIRB committee to obtain approval before conducting protocol amendment or changes in the researcher team. In addition, if the project amendment affects the participant/ volunteer, we will inform and ask for the consent from the participant every time;

..✓.. 8) All investigators will inform the SIRB committee of any protocol deviation according to the SIRB regulations within the course, and will make every effort to prevent further occurrence;

..✓.. 9) If the research may take over one year, we will submit the annual research progress report and document asking for COA extension, 30 days prior to the expiry date. If the COA is not complete within time, we will not recruit a new research subject within the study;

..✓.. 10) After the research is accomplished, we will summarize the operating procedures and submit the close-out report to the SIRB regulations within the course.

Signature...................................................... (Principal Investigator)

(.Chodchanok Vijarnsorn MD.)

Date……..….……..…………....….........

Signature...................................................... (Co-Investigator)

(.Kanokvalee Santimahakullert MD.)

Date….…………………......……...........

Signature...................................................... (Co-Investigator)

(Mr. Sappaya Krongsrattha, medical student)

Date ….………..............…....….........

Signature...................................................... (Co-Investigator)

(Yuttapong Wongswadiwat MD.)

Date ….………..............…....….........

**Suggestion and approval from the Head of Department/ Head of Division (In case of the thesis of Master’s degree and PhD students, the Head of Department/Division or Chairman of program can issue the approval)**

As the Head of Department/Division or Chairman of the Academic Program,

........... I approve and will support the research study as proposed;

........... I agree that the principal investigator and team have the knowledge, ability, potential, and are ready to conduct the research study as proposed, while protecting the rights, safety and welfare of research participants.

........... Other suggestion (if available) ..............................................................................................................................................................................................................................................................................................................................................................................................................

Signature......................................................

(Prof. jarupim Soongswang MD)

Head of department of Pediatrics, Faculty of Medicine Siriraj Hospital

Date ….………….................…....….....................
