# Supplementary material for: A retrospective cohort study of major adverse cardiac events in children affected by Kawasaki disease with coronary artery aneurysms in Thailand
Source: PLoS One. 2022 Jan 27;17(1):e0263060. doi: 10.1371/journal.pone.0263060 (PMC8794099; doi:10.1371/journal.pone.0263060)
Supplement: S1 File — (ZIP) [file pone.0263060.s003.zip › original IRB and research proposal 2018/ßíΘ IRB α═í╩╥├2 14June2018_Final_Thai language.docx]

เอกสารหมายเลข 2

**แบบขอรับการพิจารณาจากคณะกรรมการจริยธรรมการวิจัยในคน**

**คณะแพทยศาสตร์ศิริราชพยาบาล**

**ข้อมูลทั่วไปของโครงการและผู้วิจัย (Protocol identification and Investigator)**

**1.** **ชื่อโครงการวิจัย**

(ภาษาไทย) ภาวะแทรกซ้อนทางระบบหัวใจและหลอดเลือดในผู้ป่วยโรคคาวาซากิที่มีหลอดเลือดหัวใจโป่งพอง

(ภาษาอังกฤษ) Cardiac events in Kawasaki disease with coronary aneurysms revisited

**2. ชื่อหัวหน้าโครงการวิจัย** (ภาษาไทย) รศ.พญ. ชดชนก วิจารสรณ์

(ภาษาอังกฤษ) Chodchanok Vijarnsorn MD.

🗹 อาจารย์ ตำแหน่งวิชาการ..........รองศาสตราจารย์................................................................

❏ แพทย์ประจำบ้าน ❏ แพทย์ประจำบ้านต่อยอด

❏ นักศึกษา ระดับ ❏ ปริญญาตรี ❏ ปริญญาโท ❏ ปริญญาเอก

❏ บุคลากรอื่นๆ ตำแหน่ง.....................................................

วุฒิการศึกษา: ปริญญาแพทยศาสตร์บัณฑิต, วุฒิบัตรแสดงความรู้ความชำนาญในการประกอบวิชาชีพเวชกรรม สาขากุมารเวชศาสตร์, วุฒิบัตรแสดงความรู้ความชำนาญในการประกอบวิชาชีพเวชกรรม อนุสาขากุมารเวชศาสตร์ โรคหัวใจ

สังกัด: สาขาวิชาโรคหัวใจ ภาควิชากุมารเวชศาสตร์ คณะแพทยศาสตร์ศิริราชพยาบาล

สถานที่ทำงาน/ติดต่อ: สาขาวิชาโรคหัวใจ ภาควิชากุมารเวชศาสตร์ คณะแพทยศาสตร์ศิริราชพยาบาล

โทรศัพท์(ติดต่อได้ทั้งในและนอกเวลาราชการ) 081-3447015

E-mail address: cvijarnsorn@yahoo.com

(กรุณาส่งแบบประวัติ หรือ curriculum vitae ร่วมด้วย)

- 1. **ภาระงานวิจัยในความรับผิดชอบ**

ปัจจุบันผู้วิจัยมีจำนวนโครงการวิจัยภายใต้การดูแล.........3.............โครงการ คาดว่าจำนวนผู้ร่วมวิจัย/อาสาสมัครที่อยู่ในความดูแลและต้องติดตามขณะนี้รวมทั้งหมด.......3...........คน

ประสบการณ์ด้านจริยธรรมการวิจัยในคน

🗹 ผู้วิจัยเคยผ่านการอบรมด้านจริยธรรมการวิจัยในคน ปี 2561 (พร้อมแนบประกาศนียบัตรที่ผ่านการอบรมมาด้วย)

❏ผู้วิจัยเคยผ่านการอบรมการวิจัยทางคลินิกที่ดี (GCP) ปี.......... (พร้อมแนบประกาศนียบัตรที่ผ่านการอบรมมาด้วย)

(ผู้วิจัยควรมีการพัฒนาความรู้ด้านจริยธรรมการวิจัยในคนด้วยการเข้ารับการอบรม ทุก 3 ปี)

- 1. **การมีส่วนได้ส่วนเสียของผู้วิจัยกับแหล่งสนับสนุนทุนวิจัย/ยาวิจัย/เครื่องมือวิจัย** (เช่น มีหุ้นหรือญาติสายตรงมีหุ้นในแหล่งสนับสนุนทุน, เป็นที่ปรึกษา, ได้รับการสนับสนุนจากแหล่งสนับสนุนทุนเกิน 300,000 บาท/ปี เป็นต้น)

🗹 ไม่มี ❏ มี ระบุ (กรุณาแนบเอกสารหมายเลข 14)

**3. ผู้วิจัยร่วมทั้งหมด**

**3.1 ชื่อผู้วิจัยร่วม**

(ภาษาไทย) พญ. กนกวลี สันติมหกุลเลิศ (ภาษาอังกฤษ) Dr. Kanokvalee Santimahakullert

❏ อาจารย์ ตำแหน่งวิชาการ...........................................................................

❏ แพทย์ประจำบ้าน 🗹 แพทย์ประจำบ้านต่อยอด

❏ นักศึกษา ระดับ ❏ปริญญาตรี ❏ ปริญญาโท ❏ ปริญญาเอก

❏บุคลากรอื่นๆ ตำแหน่ง.....................................................

วุฒิการศึกษา: ปริญญาแพทยศาสตร์บัณฑิต, วุฒิบัตรแสดงความรู้ความชำนาญในการประกอบวิชาชีพเวชกรรม สาขากุมารเวชศาสตร์

สังกัด: สาขาวิชาโรคหัวใจ ภาควิชากุมารเวชศาสตร์ คณะแพทยศาสตร์ศิริราชพยาบาล

สถานที่ทำงาน/ติดต่อ: สาขาวิชาโรคหัวใจ ภาควิชากุมารเวชศาสตร์ คณะแพทยศาสตร์ศิริราชพยาบาล โทรศัพท์ (ติดต่อได้ทั้งในและนอกเวลาราชการ)

E-mail address:s_kanokvalee@hotmail.com

(กรุณาใส่ข้อมูลของผู้วิจัยร่วมทุกคนพร้อมส่งแบบประวัติ หรือ curriculum vitae ร่วมด้วย)

ประสบการณ์ด้านจริยธรรมการวิจัยในคนของผู้วิจัยร่วมแต่ละท่าน

🗹เคยผ่านการอบรมด้านจริยธรรมการวิจัยในคน ปี 2560 (พร้อมแนบประกาศนียบัตรที่ผ่านการอบรมมาด้วย)

❏เคยผ่านการอบรมการวิจัยทางคลินิกที่ดี (GCP) ปี.......... (พร้อมแนบประกาศนียบัตรที่ผ่านการอบรมมาด้วย)

(ผู้วิจัยควรมีการพัฒนาความรู้ด้านจริยธรรมการวิจัยในคนด้วยการเข้ารับการอบรม ทุก 3 ปี)

กรณีผู้วิจัยร่วมที่ไม่เกี่ยวข้องกับผู้ป่วย/ ข้อมูลผู้ป่วย กรุณาทำบันทึกข้อความชี้แจงเหตุผล ถึงคณะกรรมการจริยธรรมฯ เพื่อพิจารณาเป็นรายๆ ไป

- **การมีส่วนได้ส่วนเสียของผู้วิจัยร่วมกับแหล่งสนับสนุนทุนวิจัย/ยาวิจัย/เครื่องมือวิจัย** (เช่น มีหุ้นหรือญาติสายตรงมีหุ้นในแหล่งสนับสนุนทุน, เป็นที่ปรึกษา, ได้รับการสนับสนุนจากแหล่งสนับสนุนทุนเกิน 300,000 บาท/ปี เป็นต้น)

🗹 ไม่มี ❏ มี (กรุณาแนบเอกสารหมายเลข 14)

**3.2 ชื่อผู้วิจัยร่วม**

(ภาษาไทย) นศพ. สัภยา ครองศรัทธา (ภาษาอังกฤษ) Mr. Sappaya Krongsrattha

❏ อาจารย์ ตำแหน่งวิชาการ..........................................................................

❏ แพทย์ประจำบ้าน ❏ แพทย์ประจำบ้านต่อยอด

❏ นักศึกษา ระดับ ❏ปริญญาตรี ❏ ปริญญาโท ❏ ปริญญาเอก

🗹 บุคลากรอื่นๆ ตำแหน่ง...นักศึกษาแพทย์ คณะแพทยศาสตร์ศิริราชพยาบาล...................

วุฒิการศึกษา: จบมัธยมปลาย

สังกัด: กำลังศึกษาเป็นนักศึกษาแพทย์ คณะแพทยศาสตร์ศิริราชพยาบาล

สถานที่ทำงาน/ติดต่อ: คณะแพทยศาสตร์ศิริราชพยาบาล

โทรศัพท์(ติดต่อได้ทั้งในและนอกเวลาราชการ) 084-3776856

E-mail address: sappayakhrongsrattha@gmail.com

(กรุณาใส่ข้อมูลของผู้วิจัยร่วมทุกคนพร้อมส่งแบบประวัติ หรือ curriculum vitae ร่วมด้วย)

ประสบการณ์ด้านจริยธรรมการวิจัยในคนของผู้วิจัยร่วมแต่ละท่าน

🗹เคยผ่านการอบรมด้านจริยธรรมการวิจัยในคน ปี 2560 (พร้อมแนบประกาศนียบัตรที่ผ่านการอบรมมาด้วย)

❏เคยผ่านการอบรมการวิจัยทางคลินิกที่ดี (GCP) ปี.......... (พร้อมแนบประกาศนียบัตรที่ผ่านการอบรมมาด้วย)

(ผู้วิจัยควรมีการพัฒนาความรู้ด้านจริยธรรมการวิจัยในคนด้วยการเข้ารับการอบรม ทุก 3 ปี)

กรณีผู้วิจัยร่วมที่ไม่เกี่ยวข้องกับผู้ป่วย/ ข้อมูลผู้ป่วย กรุณาทำบันทึกข้อความชี้แจงเหตุผล ถึงคณะกรรมการจริยธรรมฯ เพื่อพิจารณาเป็นรายๆ ไป

- **การมีส่วนได้ส่วนเสียของผู้วิจัยร่วมกับแหล่งสนับสนุนทุนวิจัย/ยาวิจัย/เครื่องมือวิจัย** (เช่น มีหุ้นหรือญาติสายตรงมีหุ้นในแหล่งสนับสนุนทุน, เป็นที่ปรึกษา, ได้รับการสนับสนุนจากแหล่งสนับสนุนทุนเกิน 300,000 บาท/ปี เป็นต้น)

🗹 ไม่มี ❏ มี (กรุณาแนบเอกสารหมายเลข 14)

**3.3 ชื่อผู้วิจัยร่วม**

(ภาษาไทย) ผศ. นพ. ยุทธพงศ์ วงศ์สวัสดิวัฒน์ (ภาษาอังกฤษ) Yuttapong Wongswadiwat ,MD

🗹 อาจารย์ ตำแหน่งวิชาการ ผู้ช่วยศาสตราจารย์

❏ แพทย์ประจำบ้าน ❏ แพทย์ประจำบ้านต่อยอด

❏นักศึกษา ระดับ ❏ปริญญาตรี ❏ ปริญญาโท ❏ ปริญญาเอก

❏บุคลากรอื่นๆ ตำแหน่ง.....................................................

วุฒิการศึกษา: ปริญญาแพทยศาสตร์บัณฑิต, วุฒิบัตรแสดงความรู้ความชำนาญในการประกอบวิชาชีพเวชกรรม สาขากุมารเวชศาสตร์, วุฒิบัตรแสดงความรู้ความชำนาญในการประกอบวิชาชีพเวชกรรม อนุสาขากุมารเวชศาสตร์ โรคหัวใจ

สังกัด: สาขาวิชาโรคหัวใจ ภาควิชากุมารเวชศาสตร์ คณะแพทยศาสตร์มหาวิทยาลัยขอนแก่น

สถานที่ทำงาน/ติดต่อ: สาขาวิชาโรคหัวใจ ภาควิชากุมารเวชศาสตร์ คณะแพทยศาสตร์มหาวิทยาลัยขอนแก่น โทรศัพท์ (ติดต่อได้ทั้งในและนอกเวลาราชการ) 084-6018199

E-mail address: nung100@yahoo.com

(กรุณาใส่ข้อมูลของผู้วิจัยร่วมทุกคนพร้อมส่งแบบประวัติ หรือ curriculum vitae ร่วมด้วย)

ประสบการณ์ด้านจริยธรรมการวิจัยในคนของผู้วิจัยร่วมแต่ละท่าน

🗹เคยผ่านการอบรมด้านจริยธรรมการวิจัยในคน ปี 2560 (พร้อมแนบประกาศนียบัตรที่ผ่านการอบรมมาด้วย)

❏เคยผ่านการอบรมการวิจัยทางคลินิกที่ดี (GCP) ปี.......... (พร้อมแนบประกาศนียบัตรที่ผ่านการอบรมมาด้วย)

(ผู้วิจัยควรมีการพัฒนาความรู้ด้านจริยธรรมการวิจัยในคนด้วยการเข้ารับการอบรม ทุก 3 ปี)

กรณีผู้วิจัยร่วมที่ไม่เกี่ยวข้องกับผู้ป่วย/ ข้อมูลผู้ป่วย กรุณาทำบันทึกข้อความชี้แจงเหตุผล ถึงคณะกรรมการจริยธรรมฯ เพื่อพิจารณาเป็นรายๆ ไป

- **การมีส่วนได้ส่วนเสียของผู้วิจัยร่วมกับแหล่งสนับสนุนทุนวิจัย/ยาวิจัย/เครื่องมือวิจัย** (เช่น มีหุ้นหรือญาติสายตรงมีหุ้นในแหล่งสนับสนุนทุน, เป็นที่ปรึกษา, ได้รับการสนับสนุนจากแหล่งสนับสนุนทุนเกิน 300,000 บาท/ปี เป็นต้น)

🗹 ไม่มี ❏ มี (กรุณาแนบเอกสารหมายเลข 14)

**4. แหล่งทุนสนับสนุนการวิจัย (Research funding)**

🗹 ไม่มีทุน ❏อยู่ระหว่างขอทุน........................................................................(ระบุชื่อแหล่งทุน)

❏ มีทุน* ❏ รัฐ ระบุแหล่งทุน…………………………………………...…………………………………...............

❏ เอกชน ระบุแหล่งทุน…………………………………………...……………………………................

❏ NGO ระบุแหล่งทุน…………………………………………...…………………………………………

❏ อื่นๆ ระบุแหล่งทุน…………………………………………...………………….……….……………...

ที่อยู่แหล่งทุน ....................................................ชื่อผู้ประสานงานของผู้ให้ทุน.......................................................

โทรศัพท์ที่ติดต่อได้ทั้งในและนอกเวลาราชการ......................................................................................................

E-mail address: ...............................................................................................................................................

**5. สถานที่ทำวิจัย**

❏Single center ระบุ ...............................................................................................................................................

🗹 Multiple centers

🗹 เฉพาะในประเทศไทย

MOU ภายในมหาวิทยาลัยมหิดล ❏ใช่ 🗹 ไม่ใช่

MOU ระหว่าง Central Research Ethics Committee (CREC) ❏ ใช่ 🗹 ไม่ใช่

(ระบุชื่อทุกสถาบันที่ร่วมโครงการวิจัย จำนวนประชากร/อาสาสมัครที่เข้าร่วมโครงการและผลการพิจารณาของคณะกรรมการจริยธรรมการวิจัยในคนแต่ละสถาบัน): ภาควิชากุมารเวชศาสตร์ คณะแพทยศาสตร์มหาวิทยาลัยขอนแก่นร่วมวิจัยโดยมีจำนวนผู้ป่วยเข้าร่วมโครงการ 100 คน

❏ ร่วมกับต่างประเทศ

(ระบุชื่อประเทศที่ร่วมโครงการวิจัย ระบุชื่อทุกสถาบันในประเทศไทยที่ร่วมโครงการวิจัยพร้อมจำนวนประชากร/ อาสาสมัครที่เข้าร่วมโครงการและผลการพิจารณาของคณะกรรมการจริยธรรมการวิจัยในคนแต่ละสถาบันในประเทศไทย)...............................................................................................................................................................

**6. ระยะเวลาที่ทำโครงการวิจัย** ตลอดโครงการ………2….........……ปี....................เดือน

ระยะเวลาเก็บข้อมูล......1............ปี.....................เดือน

(ให้เริ่มเก็บข้อมูลหลังจากได้รับการรับรองจากคณะกรรมการจริยธรรมฯ แล้ว)

**7. โครงการนี้เป็นส่วนหนึ่งของการศึกษา: เพื่อปริญญาบัตร วุฒิบัตร หรือการศึกษาอิสระ (Independent study)**

❏ ไม่ใช่

🗹 ใช่ ระบุ

❏ งานวิจัยแพทย์ใช้ทุน/แพทย์ประจำบ้าน

🗹 งานวิจัยแพทย์ประจำบ้านต่อยอด

❏ งานวิจัยปริญญาตรี ❏ งานวิจัยปริญญาโท ❏ งานวิจัยปริญญาเอก

ผ่านการอนุมัติจากคณะกรรมการหลักสูตร (วิทยานิพนธ์)หรืออาจารย์ที่ปรึกษา 🗹 ผ่าน เมื่อวันที่ 28 พ.ย.2560 ❏ ยังไม่ผ่าน

**8. สรุปโครงร่างวิจัย**

**พร้อมกับส่งโครงร่างการวิจัยฉบับเต็ม (Full protocol/Proposal) ถ้ามี**

**8.1 หลักการและเหตุผลที่ต้องทำวิจัย (Background/Rationale)**

Kawasaki disease (KD) is an acute febrile vasculitis of unknown etiology that commonly occurs in young children. It is recognized to relate with coronary artery lesion (CAL) or coronary artery aneurysms (CAAs) in 15% to 25% if left untreated ^(^[^1-3^](#_ENREF_1)^)^. Timely initiation with intravenous immunoglobulin (IVIG) has reduced the incidence of coronary artery aneurysms defined from absolute luminal dimensions to 4-10% ^(^[^1^](#_ENREF_1)^,^ [^3-5^](#_ENREF_3)^)^. Long-term complication is determined by the initial and progression of coronary artery involvement on the current follow up. Approximately one half of patients, CAAs appear to resolve within 1 to 2 years. On the other hand, some patients had persistent aneurysms which subsequently lead to thrombosis and stenotic lesions that result in myocardial ischemia and infarction ^(^[^2^](#_ENREF_2)^)^. Aggressive management such as thromboprophylaxis and revascularization intervention might be required for complicated and selected patients ^(^[^4^](#_ENREF_4)^,^ [^6^](#_ENREF_6)^,^ [^7^](#_ENREF_7)^)^.

Since American Heart Association (AHA) published guidelines for the diagnosis, treatment, and long term management of KD in 2004 and recently in 2017, criteria for diagnosis of typical and suspicious KD have been widely recognized ^(^[^3^](#_ENREF_3)^,^ [^6^](#_ENREF_6)^)^. An algorithm ensuring captures of incomplete KD in purpose to manage the patients at risk in the effective window of therapy. This has been improved management of the acute illness in addition to acknowledgment of the care that is needed in the long term especially in adults with a previous history of KD and coronary artery aneurysms ^(^[^8^](#_ENREF_8)^)^. The latest guideline stratified patients into 5 risk levels according to their relative risk of myocardial ischemia and infarction and indicated subset each risk level using current status of coronary artery ^(^[^4^](#_ENREF_4)^,^ [^6^](#_ENREF_6)^,^ [^9^](#_ENREF_9)^)^. Serial echocardiography is recommended for patients without CAAs or with transient coronary artery dilatations normalizing within the first 6 to 8 weeks after the acute presentation of the disease ^(^[^3^](#_ENREF_3)^,^ [^4^](#_ENREF_4)^,^ [^6^](#_ENREF_6)^)^. For patients with persistent CAAs, serial myocardial stress tests are recommended in addition to regular echocardiography (risk levels III–V) ^(^[^4^](#_ENREF_4)^)^.

A large Japanese nationwide survey between 1999 and 2010 ^(^[^10^](#_ENREF_10)^)^ identified 209 patients with giant aneurysms with the 10-year survival rate of 94.3% and the total cardiac event-free rate of 0.68. Mortality rate was 5.7% (12/209) and 83.3% occurred within 1 year ^(^[^10^](#_ENREF_10)^)^. A large 2‐center retrospective study of 500 CAAs in 2,860 KD patients in US between 1979 and 2014 ^(^[^11^](#_ENREF_11)^)^reported that 75% had CAA regression within 2 years of KD episode and major adverse cardiac events (MACE) occurred in 24 patients (3 deaths, 1 orthotopic heart transplant (OHT), 6 coronary bypass graft surgery, 2 percutaneous coronary intervention, 12 findings of coronary occlusion). Lack of IVIG treatment and larger CAA size at diagnosis were associated with MACE in multivariable analysis ^(^[^11^](#_ENREF_11)^)^. In Thailand, incidence of KD was from 2.14 to 3.43 cases per 100,000 children aged 0-5 years ^(^[^12^](#_ENREF_12)^)^. Multicenter study of KD patients in 1998 – 2002 reported that 15.6% of 435 patients were resistant cases^(^[^12^](#_ENREF_12)^)^. Prevalence of incomplete KD was reported of 29% (61/208) in a single center study from Northern Thailand^(^[^13^](#_ENREF_13)^)^. These represented increased risk of CAA in Thai population. Base on the latest KD guideline, definition of CAA including dimensional z- score and risk stratification has been introduced. A long‐term outcome of CAA after treatment with IVIG however is lack in Thai population. We therefore conduct this surveillance to revisit a natural history of CAA in a cohort of KD patients and to identify factors associated with MACE in Thai population using KD database of 2 large cardiac centers (Siriraj Hospital and Khonkhan University hospital).

**References**

1. Durongpisitkul K, Gururaj VJ, Park JM, Martin CF. The prevention of coronary artery aneurysm in Kawasaki disease: a meta-analysis on the efficacy of aspirin and immunoglobulin treatment. Pediatrics. 1995;96:1057-61.

2. Kato H, Sugimura T, Akagi T, Sato N, Hashino K, Maeno Y, et al. Long-term consequences of Kawasaki disease. A 10- to 21-year follow-up study of 594 patients. Circulation. 1996;94:1379-85.

3. Newburger JW, Takahashi M, Gerber MA, Gewitz MH, Tani LY, Burns JC, et al. Diagnosis, treatment, and long-term management of Kawasaki disease: a statement for health professionals from the Committee on Rheumatic Fever, Endocarditis and Kawasaki Disease, Council on Cardiovascular Disease in the Young, American Heart Association. Circulation. 2004;110:2747-71.

4. McCrindle BW, Rowley AH, Newburger JW, Burns JC, Bolger AF, Gewitz M, et al. Diagnosis, Treatment, and Long-Term Management of Kawasaki Disease: A Scientific Statement for Health Professionals From the American Heart Association. Circulation. 2017;135:e927-e99.

5. Shah V, Christov G, Mukasa T, Brogan KS, Wade A, Eleftheriou D, et al. Cardiovascular status after Kawasaki disease in the UK. Heart. 2015;101:1646-55.

6. Newburger JW, McCrindle BW, Rowley AH, Burns JC, Bolger AF, Gewitz M, et al. Kawasaki disease: State of the art

Diagnosis, Treatment, and Long-Term Management of Kawasaki Disease: A Scientific Statement for Health Professionals From the American Heart Association. Congenit Heart Dis. 2017;12:633-5.

7. Chanthong P, Sriyoschati S, Durongpisitkul K, Soongswang J, Laohaprasitiporn D, Nana A. Coronary artery bypass graft in Kawasaki disease patients: Siriraj experience. J Med Assoc Thai. 2005;88 Suppl 8:S197-202.

8. Freeman AF, Shulman ST. Kawasaki disease: summary of the American Heart Association guidelines. Am Fam Physician. 2006;74:1141-8.

9. JSC RCo. Guidelines for medical treatment of acute Kawasaki disease: report of the Research Committee of the Japanese Society of Pediatric Cardiology and Cardiac Surgery (2012 revised version) Guidelines for diagnosis and management of cardiovascular sequelae in Kawasaki disease (JCS 2013). Digest version. Pediatr Int. 2014;56:135-58.

10. Fukazawa R, Kobayashi T, Mikami M, Saji T, Hamaoka K, Kato H, et al. Nationwide Survey of Patients With Giant Coronary Aneurysm Secondary to Kawasaki Disease 1999-2010 in Japan. Circ J. 2017;82:239-46.

11. Friedman KG, Gauvreau K, Hamaoka-Okamoto A, Tang A, Berry E, Tremoulet AH, et al. Coronary Artery Aneurysms in Kawasaki Disease: Risk Factors for Progressive Disease and Adverse Cardiac Events in the US Population. J Am Heart Assoc. 2016;5.

12. Durongpisitkul K, Sangtawesin C, Khongphatthanayopthin A, Panamonta M, Sopontammarak S, Sittiwangkul R, et al. Epidemiologic study of Kawasaki disease and cases resistant to IVIG therapy in Thailand. Asian Pac J Allergy Immunol. 2006;24:27-32.

13. Sittiwangkul R, Pongprot Y, Silvilairat S, Makonkaewkeyoon K. Clinical spectrum of incomplete Kawasaki disease in Thailand. Paediatr Int Child Health. 2013;33:176-80.

**8.2 วัตถุประสงค์ของการวิจัย (Objective)**

**-** To report major adverse cardiac events (MACE) rates in KD patients with CAAs using 2017 KD guideline definition.

**-** To assess predictors of major adverse cardiac events (MACE) in Thai population with CAAs following KD.

**8.3 ประเภทของโครงการวิจัย** (เลือกได้มากกว่าหนึ่งข้อ)

❏ Experimental biomedical / Clinical research โปรดระบุ

❏ Drug trial phase....................... ระบุชื่อยา..........................................................................................

❏ Registered drug (โปรดแนบทะเบียนยา หรือเอกสารกำกับยา)

❏ Investigational (new) drug

สถานที่เก็บยาวิจัย.................................................. ได้ลงทะเบียนกับฝ่ายเภสัชกรรม ❏ แล้ว ❏ ยัง

(กรุณาลงทะเบียนสถานที่เก็บยาวิจัยกับฝ่ายเภสัชกรรมของโรงพยาบาล ตามแบบฟอร์มการลงทะเบียนบน website ของ SIRB)

❏ Medical device trial ระบุชื่อเครื่องมือ..................................................................................................

❏ Registered device (โปรดแนบทะเบียนเครื่องมือ หรือเอกสารกำกับเครื่องมือ)

❏ Investigational (new) device

❏ Vaccine trial phase....................... ระบุชื่อ/รหัสวัคซีน.......................................................................... ❏ Registered vaccine (โปรดแนบทะเบียนวัคซีน หรือเอกสารกำกับวัคซีน)

❏ Investigational (new) vaccine

❏ Experimental procedure / intervention ระบุ.......................................................................................

❏ High risk ❏ Minimal risk

❏ Bioequivalence

❏ *In vitro* / laboratory-based study

❏ Research using repository of biological products (cells, blood, tissues, fluids, etc.)

*ระบุชนิด/ปริมาณ/จำนวน product ที่ใช้ ........................................... ........................................................

(แนบเอกสารขออนุญาตใช้ repository of biological products จากหัวหน้าภาควิชา/หน่วยงาน มาพร้อมการเสนอขอรับรอง)

❏ อื่นๆ ...................................................................................................................................................

🗹 Observation clinical research

❏ Prospective (cohort) study

❏ Case series

🗹 Retrospective (chart) review

(แนบแบบบันทึกข้อความขออนุญาตใช้เวชระเบียนจากจากหัวหน้าภาควิชา/หน่วยงาน มาพร้อมการเสนอขอรับรอง)

❏ Epidemiology research

❏ Surveillance

❏ Monitoring

❏ อื่นๆ ระบุ ........................................... ................................................................................................

❏ Social / Behavioral research

❏ Questionnaire-based research

❏ อื่นๆ ระบุ .........................................................

**8.4 การออกแบบการวิจัย (Research design)**

❏ Randomized-controlled trial

❏ Quasi-experimental study (manipulation and control only, without randomization)

❏ Pre-experimental study (manipulation only, without control and randomization)

❏ Prospective cohort study

🗹 Descriptive study

🗹 Cross-sectional study

❏ Pilot study

❏อื่นๆ ระบุ.......................................................................................

**8.5 ผู้ร่วมวิจัย/อาสาสมัคร (Research subjects)**

**การคำนวณขนาดตัวอย่าง (Sample size calculation)**

จากการทบทวนวรรณกรรมที่เกี่ยวข้องพบว่า ในกลุ่มผู้ป่วยโรคคาวาซากิที่มีหลอดเลือดหัวใจโป่งพองร่วมด้วย พบเป็น 17% ของผู้ป่วยคาวาซากิทั้งหมด ^(11)^

คำนวณกลุ่มประชากรได้ดังนี้


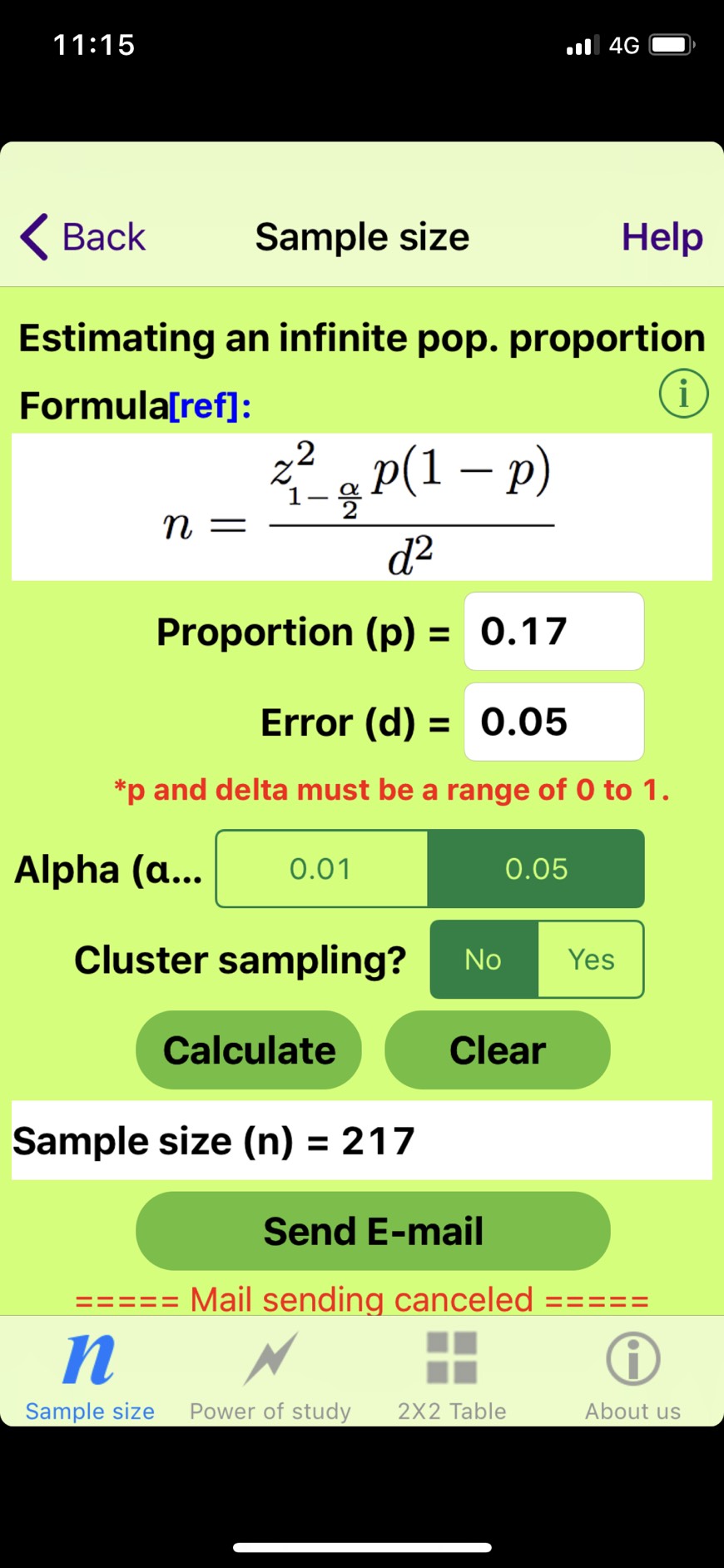


**เกณฑ์การคัดเลือกผู้ร่วมวิจัย/อาสาสมัคร (Inclusion criteria)**

1. Patients who previously diagnosed of Kawasaki disease complicated with coronary artery aneurysms in two centers (Siriraj Hospital and Khonkhan University Hospital) between 2007 and 2017

2. Patients who diagnosed of Kawasaki disease coronary artery aneurysm and clinically follow up at least a year

**เกณฑ์การคัดออกผู้ร่วมวิจัย/อาสาสมัคร (Exclusion criteria)**

Patients with incomplete initial echocardiographic data.

Patients with co-morbidity of other cardiovascular diseases such as congenital heart defect

**เกณฑ์การถอนผู้ร่วมวิจัย/อาสาสมัครหรือยุติการเข้าร่วมการวิจัย (Withdrawal or termination criteria)**

N/A as a retrospective trial.

**การจัดผู้ร่วมวิจัย/อาสาสมัครเข้ากลุ่ม (Subject allocation)**

N/A

**8.6 กระบวนการวิจัย** ระบุรายละเอียดของกระบวนการวิจัย เครื่องมือที่ใช้ในการวิจัย ขั้นตอนต่างๆ ในการดำเนินการวิจัย สิ่งที่ผู้ร่วมวิจัย/อาสาสมัครจะต้องปฏิบัติหรือจะได้รับการปฏิบัติ (เช่น จำนวนครั้งที่มีการเจาะเลือด ปริมาณเลือดที่เจาะ จำนวนการนัดหมาย และเวลาที่ใช้ในการร่วมวิจัย เป็นต้น) หากมีการส่งโครงการวิจัยแนบมาด้วย ขอให้มีใจความที่ตรงกัน ทั้งฉบับภาษาไทยและภาษาอังกฤษ รวมถึงอ้างอิงเลขหน้าในโครงการวิจัยในส่วนที่เกี่ยวข้อง

Data Collection**:** (please see CRF)

Pertinent data will incorporate the following parameters:

1. Demographic information: age, gender, date of birth, initial diagnosis, initial weight, height, BSA

2. Clinical data: date of diagnosis, presentation at initial diagnosis, initial laboratory including; ESR, CRP, CBC, ALT, albumin, concomittent infection, presence of heart failure, cardiogenic shock,

3. Echocardiographic assessment:

Initial echocardiography: date of echocardiography, LVEF, presence of LV systolic function impairment, initial coronary involvement,

Echo Dx CAAs: date of diagnosis of CAAs, LVEF, presence of LV systolic function impairment, initial coronary involvement,

Echo at 8 weeks post diagnosis: progression

Echo at 1 year post KD: progression

4. Treatment: date of IVIg, receiving 2^nd^ dosage IVIg, date of repeat IVIg, adjunctive anti-inflammatory medications

5. Other tests:

- CAG findings, coronary stenosis present or not

- Stress MPI assessment: type, findings

6. Clinical outcomes in 1 year post KD including major adverse cardiac events: date of 1 year follow up, NYHA, coronary intervention, cardiac death, medications, date of surgical procedure, intervention procedure, date of intervention procedure, current cardiac problem; symptoms; chest pain, syncope, arrhythmia

7. Recent clinical outcomes in 1 year post KD including major adverse cardiac events: date of 1 year follow up, NYHA, coronary intervention, cardiac death, medications, date of surgical procedure, intervention procedure, date of intervention procedure, current cardiac problem; symptoms; chest pain, syncope, arrhythmia, recent risk level, nutritional status, recent lipid profile, recent blood pressure, smoking, co-morbid.

Definition of CAA and MACE (also presented in the last page of CRF)

| Items | Definition |
| --- | --- |
| Late diagnosis and treated KD | KD which initiated treatment at >= 10 days of onset of fever  *Ref: AHA KD guideline 2017* |
| Coronary involvement and CAA criteria (echo, CAG) | Using Z-Score Classification  1. No involvement: Always <2  2. Dilation only: 2 to <2.5; or if initially <2, a decrease in Z score during follow-up ≥1  3. Small aneurysm: 2.5 to <5  4. Medium aneurysm: 5 to <10, and absolute dimension <8 mm  5. Large or giant aneurysm: > 10, or absolute dimension >8 mm  Remarks: One potential limitation of this study is that regression formulas for the LAD were used to derive Z scores for the left circumflex branch (normal values for the circumflex are not available with the Z-score system that was used).  *Ref: AHA KD guideline 2017* |
| Risk level of KD (based on 2017 KD guideline) | 1: No involvement,  2: Dilation only  3.1: Small aneurysm, current or persistent  3.2: Small aneurysm, regressed to normal or dilation only  4.1: Medium aneurysm, current or persistent  4.2: Medium aneurysm, regressed to small aneurysm  4.3: Medium aneurysm, regressed to normal or dilation only  5.1: Large and giant aneurysm, current or persistent  5.2: Large or giant aneurysm, regressed to medium aneurysm  5.3: Large or giant aneurysm, regressed to small aneurysm  5.4: Large or giant aneurysm,regressed to normal or dilation only  *Ref: AHA KD guideline 2017* |
| MACE (major adverse cardiac events | All-cause mortality or re-hospitalization for a cardiovascular-related illness. Cardiovascular-related illnesses included heart failure, reinfarction (nonfatal), recurrence of angina pectoris and repeat PCI or CABG  *Ref :*  *Califf RM, Bengtson JR. Cardiogenic shock. N Engl J Med. 1994;330:1724–30.*  *Sai IT, Wang CP, Lu YC, et al. BMC Cardiovasc Disord. 2017 Jan 4;17(1):1.* |

.......................................................................................................................................................................................

**8.7 กระบวนการเก็บข้อมูล (Data collection process)** กรุณาส่งแบบบันทึกการเก็บข้อมูล (case record form) และ/หรือ แบบสอบถาม (questionnaire) และ/หรือ แบบสัมภาษณ์ (interview question) และ/หรือ บทสัมภาษณ์ทางโทรศัพท์ (telephone script) ที่จะใช้มาประกอบการพิจารณาด้วย (ถ้ามี)*

- ได้แนบแบบบันทึกข้อมูล,ของผู้ร่วมวิจัย/อาสาสมัคร โดยใช้เป็นรหัสแทนการระบุชื่อ-นามสกุล,Hospital Number(HN) หรือ identification รูปแบบอื่นๆที่สามารถระบุผู้ร่วมวิจัย/อาสาสมัครได้เป็นรายบุคคล

**ผู้วิจัยจะเริ่มเก็บข้อมูลหลังจากได้รับรองจากคณะกรรมการจริยธรรมการวิจัยในคนแล้ว**

.......................................................................................................................................................................................

**8.8 การวัดผล/การวิเคราะห์ผลการวิจัย (Outcome measurement / Data analysis)** (กระบวนการวัดผล/วิเคราะห์ผล

รวมถึงสถิติที่ใช้)

Patients’ baseline characteristics will be summarized using descriptive statistic presenting in percentage, mean and standard deviation. CMR results will be correlated to epicardial vessels patency. The association of cardiac events and perfusion defect will be determined by risk analysis**.**

**8.9 หลักฐาน ข้อมูล หรือเอกสารอ้างอิง** (วิธีการเขียนเอกสารอ้างอิงให้เขียนตามาตรฐานสากล)

**References**

1. Durongpisitkul K, Gururaj VJ, Park JM, Martin CF. The prevention of coronary artery aneurysm in Kawasaki disease: a meta-analysis on the efficacy of aspirin and immunoglobulin treatment. Pediatrics. 1995;96:1057-61.

2. Kato H, Sugimura T, Akagi T, Sato N, Hashino K, Maeno Y, et al. Long-term consequences of Kawasaki disease. A 10- to 21-year follow-up study of 594 patients. Circulation. 1996;94:1379-85.

3. Newburger JW, Takahashi M, Gerber MA, Gewitz MH, Tani LY, Burns JC, et al. Diagnosis, treatment, and long-term management of Kawasaki disease: a statement for health professionals from the Committee on Rheumatic Fever, Endocarditis and Kawasaki Disease, Council on Cardiovascular Disease in the Young, American Heart Association. Circulation. 2004;110:2747-71.

4. McCrindle BW, Rowley AH, Newburger JW, Burns JC, Bolger AF, Gewitz M, et al. Diagnosis, Treatment, and Long-Term Management of Kawasaki Disease: A Scientific Statement for Health Professionals From the American Heart Association. Circulation. 2017;135:e927-e99.

5. Shah V, Christov G, Mukasa T, Brogan KS, Wade A, Eleftheriou D, et al. Cardiovascular status after Kawasaki disease in the UK. Heart. 2015;101:1646-55.

6. Newburger JW, McCrindle BW, Rowley AH, Burns JC, Bolger AF, Gewitz M, et al. Kawasaki disease: State of the art

Diagnosis, Treatment, and Long-Term Management of Kawasaki Disease: A Scientific Statement for Health Professionals From the American Heart Association. Congenit Heart Dis. 2017;12:633-5.

7. Chanthong P, Sriyoschati S, Durongpisitkul K, Soongswang J, Laohaprasitiporn D, Nana A. Coronary artery bypass graft in Kawasaki disease patients: Siriraj experience. J Med Assoc Thai. 2005;88 Suppl 8:S197-202.

8. Freeman AF, Shulman ST. Kawasaki disease: summary of the American Heart Association guidelines. Am Fam Physician. 2006;74:1141-8.

9. JSC RCo. Guidelines for medical treatment of acute Kawasaki disease: report of the Research Committee of the Japanese Society of Pediatric Cardiology and Cardiac Surgery (2012 revised version) Guidelines for diagnosis and management of cardiovascular sequelae in Kawasaki disease (JCS 2013). Digest version. Pediatr Int. 2014;56:135-58.

10. Fukazawa R, Kobayashi T, Mikami M, Saji T, Hamaoka K, Kato H, et al. Nationwide Survey of Patients With Giant Coronary Aneurysm Secondary to Kawasaki Disease 1999-2010 in Japan. Circ J. 2017;82:239-46.

11. Friedman KG, Gauvreau K, Hamaoka-Okamoto A, Tang A, Berry E, Tremoulet AH, et al. Coronary Artery Aneurysms in Kawasaki Disease: Risk Factors for Progressive Disease and Adverse Cardiac Events in the US Population. J Am Heart Assoc. 2016;5.

12. Durongpisitkul K, Sangtawesin C, Khongphatthanayopthin A, Panamonta M, Sopontammarak S, Sittiwangkul R, et al. Epidemiologic study of Kawasaki disease and cases resistant to IVIG therapy in Thailand. Asian Pac J Allergy Immunol. 2006;24:27-32.

13. Sittiwangkul R, Pongprot Y, Silvilairat S, Makonkaewkeyoon K. Clinical spectrum of incomplete Kawasaki disease in Thailand. Paediatr Int Child Health. 2013;33:176-80.

**ข้อพิจารณาด้านจริยธรรมการวิจัยในคน (Ethical consideration)**

**9. ลักษณะผู้ร่วมวิจัย/อาสาสมัคร**

❏ Healthy volunteers

❏ Patients excluding vulnerable subjects

🗹 อื่นๆ เช่น Retrospective chart review

❏ Vulnerable subjects* ระบุ (ผู้ที่ไม่สามารถรับรู้ ไม่สามารถตัดสินใจหรือไม่มีอิสระในการตัดสินใจด้วยตัวเอง)

❏ เด็กเล็ก / ผู้ที่ยังไม่บรรลุนิติภาวะ (อายุ <18 ปี)**

❏ ผู้พิการหรือมีความบกพร่องทางสมอง / จิตใจ

❏ ผู้ป่วยห้องฉุกเฉิน หรือ หออภิบาลผู้ป่วยหนัก, ผู้ป่วยระยะสุดท้าย

❏ ผู้ป่วยเรื้อรังที่ต้องพึ่งพาแพทย์และผู้ดูแล

❏ หญิงมีครรภ์ ❏ นักเรียน / นักศึกษา ❏ นักโทษ

❏ ทหาร ผู้ใต้บังคับบัญชา ❏ ผู้อยู่ตามสถานสงเคราะห์ ❏ ผู้อ่านหนังสือไม่ออก

❏ อื่นๆ ระบุ ........................................... ..........................................................................................

*ถ้ามีผู้ร่วมวิจัย/อาสาสมัครในกลุ่มนี้ หากจะต้องขอความยินยอมจากผู้แทนโดยชอบธรรม ผู้วิจัยคาดว่าจะขอความยินยอมจากผู้ใด โปรดระบุ....................................................................................................................................................

**ในกรณีเด็ก อายุ 7-12 ปี **อาจ**ต้องมีการขอความยินยอมจากเด็กโดยตรง (assent) เพิ่มเติมจากการขอความยินยอมจากผู้ปกครองหรือผู้แทนโดยชอบธรรม (consent)

**ในกรณีเด็ก อายุ 12- น้อยกว่า18 ปี **ต้อง**มีการขอความยินยอมจากเด็กโดยตรง (assent) เพิ่มเติมจากการขอความยินยอมจากผู้ปกครองหรือผู้แทนโดยชอบธรรม (consent) (ยกเว้นบางกรณี เช่น เด็กสติปัญญาบกพร่อง เป็นต้น)

**10. การใช้ข้อมูลและการเก็บชีววัตถุของผู้ร่วมวิจัย/อาสาสมัคร**

**10.1 มีการขออนุญาตใช้** **repository of biological products จากผู้มีอำนาจ** 🗹 ไม่มี ❏ มี

**10.2 มีการขอเก็บชีววัตถุของผู้ร่วมวิจัย/อาสาสมัครไว้เพื่อศึกษาต่อในอนาคต** 🗹 ไม่มี ❏ มี

**10.3 มีการส่ง Specimen ออกนอกสถาบัน** 🗹 ไม่มี ❏ มี

**มีการนำ Specimen จากภายนอกเข้ามาในสถาบัน** 🗹 ไม่มี ❏ มี

(ข้อ 10.3 ทั้ง 2 กรณี ต้องทำ Material Transfer Agreement และต้องส่งสำเนาเอกสารให้คณะกรรมการจริยธรรมการวิจัยในคนเมื่อดำเนินการเรียบร้อยแล้ว มิฉะนั้นผู้วิจัยจะไม่สามารถรับเอกสารรับรอง (COA) จากคณะกรรมการจริยธรรมฯ)

**11. กระบวนการเชิญชวนให้เข้าร่วมการวิจัย (Recruitment process)**

N/A เนื่องจากเป็น retrospective chart review

**12. กระบวนการขอความยินยอมให้เข้าร่วมการวิจัย (Informed consent process)**

🗹ไม่เกี่ยวข้องเนื่องจากเป็น Retrospective chart review

❏ ขอยกเว้นกระบวนขอความยินยอม (แนบเอกสารหมายเลข 1จ)

❏ขอความยินยอมให้ระบุข้อมูลต่อไปนี้

- ผู้ดำเนินการขอความยินยอม
- ระยะเวลาในการให้คำแนะนำกลุ่มผู้จะเชิญเข้าโครงการ เพื่อขอความยินยอมเข้าร่วมโครงการ.......................

ผู้เข้าร่วมการวิจัยมีโอกาสที่จะนำเอกสารชี้แจงฯ กลับไปทบทวนก่อนการตัดสินใจหรือไม่....................................

- ภาษาที่ใช้ประจำของผู้ที่จะเชิญเข้าโครงการ หรือผู้แทนโดยชอบธรรม........................
- ภาษาที่ใช้ในการขอความยินยอม ..............................................................................
- การดูแลเรื่องความเป็นส่วนตัว และการรักษาความลับ ❏ สถานที่ ..............................

**12.1 เอกสารที่เกี่ยวข้อง** (ท่านสามารถเลือกทุกข้อที่เกี่ยวข้อง)

❏เอกสารชี้แจงผู้ร่วมวิจัย/อาสาสมัคร (เอกสารหมายเลข 3ก) และ ❏ หนังสือแสดงเจตนายินยอมเข้าร่วมการวิจัยโดยได้รับการบอกกล่าวและเต็มใจ (เอกสารหมายเลข 3ข)

❏สำหรับเด็กอายุ 7-12 ปี เอกสารชี้แจงผู้เข้าร่วมการวิจัยและแสดงความสมัครใจเข้าร่วมโครงการวิจัยสำหรับเด็ก (assent form) (เอกสารหมายเลข 4.1) พร้อมแนบเอกสารชี้แจงและหนังสือแสดงความยินยอมจากผู้ปกครอง/ผู้แทนโดยชอบธรรม (เอกสารหมายเลข 3ก และ 3ข โดยปรับสรรพนามให้เหมาะสม)

❏สำหรับเด็กอายุมากกว่า 12 ปีถึงน้อยกว่า 18 ปี เอกสารชี้แจงผู้เข้าร่วมการวิจัยและแสดงความสมัครใจเข้าร่วมโครงการวิจัยสำหรับเด็ก (assent form) (เอกสารหมายเลข 4.2) พร้อมแนบเอกสารชี้แจงและหนังสือแสดงความยินยอมจากผู้ปกครอง/ผู้แทนโดยชอบธรรม (เอกสารหมายเลข 3ก และ 3ข โดยปรับสรรพนามให้เหมาะสม)

❏ผู้ที่ไม่สามารถให้ความยินยอมได้ อื่นๆ ระบุ ...........................พร้อมแนบเอกสารชี้แจงและหนังสือแสดงความ

ยินยอมจากผู้ปกครอง/ผู้แทนโดยชอบธรรม (เอกสารหมายเลข 3ก และ 3ข โดยปรับสรรพนามให้เหมาะสม)

**12.2 กระบวนการขอความยินยอมจากอาสาสมัครและ/หรือผู้แทนโดยชอบธรรม**

**12.2.1.** **ผู้ทำหน้าที่ขอความยินยอมอาสาสมัครเข้าร่วมโครงการวิจัย (ท่านสามารถเลือกทุกข้อที่เกี่ยวข้อง)**

❏ หัวหน้าโครงการวิจัย ❏ ผู้วิจัยร่วม ❏ ผู้ช่วยโครงการวิจัย (เช่น research nurse, นักศึกษา)

❏ แพทย์เจ้าของไข้ ❏ อื่นๆ ระบุ............................................................................................

**12.2.2. อธิบายกระบวนการขอความยินยอมจากผู้ร่วมวิจัย/อาสาสมัครหรือผู้แทนโดยชอบธรรมอย่างละเอียด**

……………………………………………………………………………………………………………………………………………..………………….

* ในกรณีที่ขอความยินยอมจากผู้แทนโดยชอบธรรมถ้าผู้ร่วมวิจัย/อาสาสมัครกลับมาอยู่ในสภาวะที่สามารถให้ความยินยอมด้วยตนเองได้ให้ขอความยินยอมใหม่

**13. ประโยชน์ที่คาดว่าจะได้รับจากการวิจัย**

13.1 ประโยชน์ต่อผู้ร่วมวิจัย/อาสาสมัครเป็นรายบุคคล – โครงงานวิจัยนี้เป็น observational study ที่มีการติดตามผู้ป่วยระยะยาว ไม่มีผลต่อผู้ป่วยโดยตรง แต่ทำให้ทราบอาการล่าสุดของผู้ป่วย และอาจสามารถสืบค้นเพิ่มเติมกรณีมีข้อบ่งชี้ อย่างไรก็ตามผลการวิจัยจะสามารถนำมาประยุกต์ใช้ในการพัฒนาการรักษาผู้ป่วยกลุ่มนี้ในอนาคต…….……

13.2 ประโยชน์ต่อวิชาชีพโดยรวม - ผลการวิจัยจะสามารถนำมาประยุกต์ใช้ในการพัฒนาการรักษาผู้ป่วยกลุ่มนี้ในอนาคต

13.3 ประโยชน์ต่อสังคม- ผลการวิจัยจะสามารถนำมาประยุกต์ใช้ในการพัฒนาการรักษาผู้ป่วยกลุ่มนี้ในอนาคต

13.4 อื่น ๆ ……………………………………….………………………….……………………………..…….................

**14. ผลกระทบที่อาจจะเกิดแก่ผู้ร่วมวิจัย/อาสาสมัคร และการชดเชย**

14.1 อธิบายผลกระทบต่อร่างกาย จิตใจ สังคม เศรษฐกิจ โดยเฉพาะความเสี่ยงมีหรือไม่ (เคยมีการวิจัยทำนองเดียวกับโครงร่างที่เสนอนี้มาก่อนหรือไม่ และเคยเกิดเหตุการณ์ไม่พึงประสงค์อย่างไร กรุณาระบุรายละเอียดและโอกาสที่อาจเกิดเหตุการณ์ไม่พึงประสงค์จากข้อมูลที่มี หรือจากการประเมินของผู้วิจัย) รวมถึงความไม่สะดวกสบาย และการเสียเวลา

- ไม่มีเนื่องจากเป็น observational study

14.2 มาตรการป้องกันและแก้ไขเมื่อเกิดเหตุการณ์ไม่พึงประสงค์ที่ผู้วิจัยเตรียมไว้ในโครงการนี้

- ไม่มีเนื่องจากเป็น observational study

14.3 ผู้รับผิดชอบค่าใช้จ่าย/ค่าชดเชยในการแก้ไขหรือรักษาเหตุการณ์ไม่พึงประสงค์จากการวิจัย หากมีการจัดหาประกันต่อความเสียหาย/บาดเจ็บ ให้แนบใบรับรองและสำเนากรมธรรม์ *(หากผู้วิจัยเป็นบุคลากรสังกัดคณะฯ และไม่ได้รับทุนจากหน่วยงานเอกชนภายนอกสามารถระบุโรงพยาบาลศิริราชเป็นผู้รับผิดชอบกรณีเกิดเหตุการณ์ไม่พึงประสงค์จากการวิจัย)*

- ไม่มีเนื่องจากเป็น observational study

14.4 ค่าใช้จ่ายที่ผู้ร่วมวิจัย/อาสาสมัครต้องรับผิดชอบเองและค่าตอบแทนที่จะได้รับ

- ไม่มีเนื่องจากเป็น observational study

14.5 ชื่อผู้รับผิดชอบหรือแพทย์ และหมายเลขโทรศัพท์ที่สามารถติดต่อได้ตลอดเวลาหากเกิดเหตุการณ์ไม่พึงประสงค์จากการวิจัย

- รศ.พญ. ชดชนก วิจารสรณ์ 081-3447015

14.6 กรณีเป็นการวิจัยทางคลินิก การวิจัยที่ใช้ผลตรวจต่างๆ เช่น ผลตรวจทางห้องปฏิบัติการ ผลตรวจทางพยาธิวิทยา ผลตรวจทางรังสี เป็นต้น ผู้วิจัยมีวิธีการอย่างไรในการแจ้งแพทย์เจ้าของไข้ หรือ แพทย์อื่นๆ ที่เป็นผู้ให้การรักษาผู้ร่วมวิจัย/อาสาสมัครให้ทราบว่าบุคคลผู้นั้นอยู่ในระหว่างการเข้าร่วมการวิจัย

- แจ้งในที่ประชุมสาขาวิชา วันที่ 19 ตุลาคม 2560 แพทย์เจ้าของไข้รับทราบการทำ observational study

หมายเหตุ: กรณีโครงการวิจัยที่มี intervention ต่อผู้เข้าร่วมการวิจัย ขอให้ผู้วิจัย ลงข้อมูล รหัสโครงการ ชื่อหัวหน้าโครงการ และหมายเลขโทรศัพท์ไว้ในแฟ้มประวัติผู้ป่วย

14.7 โครงการวิจัยมีคณะกรรมการติดตามดูแล เช่น Study monitoring, Data Safety Monitoring Board (DSMB) เป็นต้น

❏ มี ระบุ...........................................................................

❏ ไม่มี 🗹 ไม่เกี่ยวข้อง

14.8 ทางเลือกอื่นๆ ในการดูแลรักษา

❏ มี ระบุ...........................................................................

❏ ไม่มี 🗹 ไม่เกี่ยวข้อง

14.9 โครงการวิจัยมีแผนที่จะทำการวิเคราะห์ระหว่างดำเนินการ (Interim analysis) ในแง่ของความเสี่ยงของทั้งโครงการ

❏ มี ระบุเมื่อไหร่...............................................................

❏ ไม่มี 🗹 ไม่เกี่ยวข้อง

**15. เกี่ยวข้องหรืออาจมีผลกระทบกระเทือนต่อศาสนา ความเชื่อ ขนบธรรมเนียมประเพณีหรือวัฒนธรรมอันดีงาม ชื่อเสียงของสถาบัน ท้องถิ่นหรือประเทศที่ทำการวิจัยอยู่หรือไม่**

❏ เกี่ยวข้อง ระบุวิธีการป้องกันหรือลดผลกระทบดังกล่าว.................................................. 🗹 ไม่เกี่ยวข้อง

**16. วิธีการปกป้องความลับหรือข้อมูลส่วนตัวของผู้ร่วมวิจัย/อาสาสมัคร** (🗹 ทุกข้อที่เกี่ยวข้อง)

16.1 ก. มีสถานที่เหมาะสม เป็นสัดส่วน เฉพาะในการขอความยินยอม

🗹 มี (ระบุ สาขาวิชาโรคหัวใจ กุมารเวชศาสตร์ รพ.ศิริราช, รพ.ศรีนครินทร์ขอนแก่น) ❏ ไม่มี

ข. มีสถานที่เหมาะสม เป็นสัดส่วนในการดำเนินการศึกษาวิจัย

🗹 มี (ระบุ สาขาวิชาโรคหัวใจ กุมารเวชศาสตร์ รพ.ศิริราช, รพ.ศรีนครินทร์ขอนแก่น) ❏ ไม่มี

16.2 วิธีการบันทึกข้อมูลส่วนตัว

❏ ไม่มีการบันทึกข้อมูลส่วนตัวของผู้ร่วมวิจัย/อาสาสมัคร

🗹มีการบันทึกข้อมูลส่วนตัวของผู้ร่วมวิจัย/อาสาสมัคร (ต้องตอบข้อ 16.3 ด้วย)

ให้ใช้รหัสแทนชื่อและข้อมูลส่วนตัวของผู้ร่วมวิจัย/อาสาสมัคร ไม่ระบุวันเดือนปีเกิด อักษรตัวแรกของชื่อ นามสกุล

🗹 เป็นไฟล์อิเลกทรอนิกส์ ❏ รูปถ่าย / ภาพนิ่ง ❏ วิดิทัศน์ / ภาพเคลื่อนไหว

❏ บันทึกเสียง ❏ อื่นๆ ระบุ ...........................................................................................

16.3 หากมีการบันทึกข้อมูลส่วนตัวดังกล่าวข้างต้น โปรดระบุผู้ที่สามารถเข้าถึงข้อมูลได้ วิธีการป้องกันบุคคลที่ไม่เกี่ยวข้องในการเข้าถึงข้อมูล ระบุระยะเวลาในการเก็บข้อมูลไว้ และวิธีการทำลายข้อมูลเมื่อสิ้นสุดการวิจัย

🗹 บันทึกไว้ในคอมพิวเตอร์ส่วนตัวที่มีรหัสป้องกันบุคคลอื่นไม่ให้สามารถเปิดได้

❏ เก็บเอกสาร/แผ่น CD / ไฟล์ ในตู้/ลิ้นชัก ที่มีกุญแจล็อก และผู้วิจัยเท่านั้นที่มีกุญแจเปิด-ปิด

🗹 มีการทำลายเอกสาร / CD / ไฟล์ ทั้งหมดเมื่อสิ้นสุดการวิจัย

❏ ส่งแผ่น CD ประวัติผู้ป่วยคืนงานเวชระเบียนเมื่อสิ้นสุดการวิจัย

❏ เก็บเอกสาร/แผ่น CD / ไฟล์ ไว้ต่อเป็นเวลา........ปี หลังสิ้นสุดการวิจัย

❏ อื่นๆ ระบุ ........................................................................................................

ระบุผู้เข้าถึงข้อมูล………………………………………………………………………………………………………..

หัวหน้าโครงการวิจัยเป็นผู้รับผิดชอบในการรักษาความลับผู้ร่วมวิจัย/อาสาสมัคร และต้องแจ้งไว้ในเอกสารชี้แจงผู้ร่วมวิจัย/อาสาสมัคร

**17. คำรับรองของผู้วิจัย**

*(ขอให้ผู้วิจัยหลักและผู้วิจัยร่วม*ทุกคน*ที่ระบุไว้ในข้อ 2 และ3 พิจารณาแนวทางดำเนินการวิจัยตามคำรับรองด้านล่างนี้*

*แล้วกาเครื่องหมายหน้าข้อ พร้อมลงนามและระบุวันที่ในตอนท้าย เพื่อรับรองแนวทางดำเนินการวิจัย)*

.. 🗹...... 1) ข้าพเจ้าและคณะผู้วิจัยดังมีรายนามและได้ลงชื่อไว้ในเอกสารนี้ จะประพฤติปฏิบัติตามจรรยาบรรณของนักวิจัย เพื่อให้การดำเนินงานวิจัยตั้งอยู่บนพื้นฐานตามหลักจริยธรรมการวิจัยในคนและหลักวิชาการที่เหมาะสม ตลอดจนประกันมาตรฐานของการศึกษาค้นคว้าให้เป็นไปอย่างสมศักดิ์ศรีและเกียรติภูมิของนักวิจัย

.. 🗹...... 2) ข้าพเจ้าและคณะผู้วิจัย จะดำเนินงานวิจัยตามที่ระบุไว้ในโครงร่างการวิจัยฉบับที่ได้รับการรับรองจากคณะกรรมการจริยธรรมการวิจัยในคนโดยเคร่งครัด พร้อมใช้เอกสารชี้แจง หนังสือแสดงเจตนายินยอมจากผู้เข้าร่วมวิจัย และเอกสารอื่นๆ ที่ได้ผ่านการรับรองจากคณะกรรมการจริยธรรมฯ แล้วเท่านั้น

.. 🗹...... 3) ข้าพเจ้าและคณะผู้วิจัย มีความรู้ความเข้าใจในกระบวนการวิจัยที่เสนอมาอย่างดีทุกขั้นตอน และมีความสามารถในการแก้ไขปัญหา หรือเหตุการณ์ไม่พึงประสงค์ที่อาจจะเกิดขึ้นในระหว่างการวิจัย โดยคำนึงถึงศักดิ์ศรี สิทธิ ความปลอดภัย และสวัสดิภาพของผู้เข้าร่วมวิจัยเป็นสำคัญ

.. 🗹...... 4) ข้าพเจ้าและคณะผู้วิจัยจะไม่เริ่มดำเนินการวิจัย จนกว่าจะได้รับเอกสารรับรอง (Certificate of Approval, COA) จากคณะกรรมการจริยธรรมฯ แล้วเท่านั้น

.. 🗹...... 5) ข้าพเจ้าและคณะผู้วิจัยเข้าใจดีในการเข้าถึงข้อมูล และจะจัดการปกป้องความลับข้อมูลของผู้เข้าร่วมวิจัยอย่างเคร่งครัด ผู้เข้าร่วมวิจัยสามารถเชื่อมั่นได้ว่าข้อมูลที่ได้เปิดเผยต่อคณะผู้วิจัยจะถูกเก็บเป็นความลับ

.. 🗹...... 6) ข้าพเจ้าจะรายงานเหตุการณ์ไม่พึงประสงค์รุนแรง /เหตุการณ์ที่ไม่สามารถคาดเดาได้ล่วงหน้าในระหว่างการวิจัย (serious / unexpected adverse event) ตามระเบียบของคณะกรรมการจริยธรรมฯ ภายในเวลาที่กำหนด และจะให้ความช่วยเหลือในการแก้ไขเหตุการณ์ไม่พึงประสงค์ที่เกิดขึ้นระหว่างการวิจัยอย่างเต็มความสามารถ

.. 🗹...... 7) หากมีความจำเป็นต้องปรับแก้ไขโครงการวิจัย (protocol amendment) หรือมีการเปลี่ยนแปลงคณะผู้วิจัย ข้าพเจ้าจะแจ้งคณะกรรมการจริยธรรมฯ เพื่อขอการรับรองก่อนเริ่มดำเนินการตามที่ต้องการปรับเปลี่ยนทุกครั้ง และหากการปรับโครงการวิจัยมีผลกระทบต่อผู้เข้าร่วมวิจัย ข้าพเจ้าจะแจ้งและขอความยินยอมจากผู้ที่เข้าร่วมการวิจัยแล้วอีกครั้ง

.. 🗹...... 8) ข้าพเจ้าจะรายงานการดำเนินการใดๆที่ไม่เป็นไปตามที่ระบุไว้ในโครงร่างการวิจัย (protocol deviation) ตามระเบียบของคณะกรรมการจริยธรรมฯ ภายในเวลาที่กำหนด และจะหาทางป้องกันมิให้เกิดซ้ำอีก อย่างเต็มความสามารถ

.. 🗹...... 9) หากการวิจัยดำเนินการไม่เสร็จสิ้นใน 1 ปี ข้าพเจ้าจะรายงานความคืบหน้าของโครงการอย่างน้อยทุก 1 ปี (progress report) และข้าพเจ้ามีหน้าที่ขอต่ออายุเอกสารรับรองจากคณะกรรมการจริยธรรมฯ ภายใน 30 วันก่อนครบกำหนด (COA extension) ทั้งนี้หากอายุเอกสารรับรองขาดช่วง จะไม่มีการรับผู้เข้าร่วมวิจัยใหม่จนกว่าการต่ออายุจะเสร็จเรียบร้อย

..🗹..... 10) เมื่อทำการวิจัยเสร็จสิ้น ข้าพเจ้าจะสรุปรายงานผลการดำเนินการ เพื่อแจ้งปิดโครงการวิจัย (close out report) ตามระเบียบของคณะกรรมการจริยธรรมฯ ภายในเวลาที่กำหนด

ลงชื่อ ......................................................หัวหน้าโครงการวิจัย (Principle Investigator)

(รศ.พญ.ชดชนก วิจารสรณ์)

วันที่ ….…/………..............…/....….........

ลงชื่อ ...................................................... ผู้วิจัยร่วม (Co-Investigator)

(พญ.กนกวลี สันติมหกุลเลิศ)

วันที่ ….…/………..............…/....….........

ลงชื่อ ...................................................... ผู้วิจัยร่วม (Co-Investigator)

(นศพ. สัภยา ครองศรัทธา)

วันที่ ….…/………..............…/....….........

ลงชื่อ ...................................................... ผู้วิจัยร่วม (Co-Investigator)

(ผศ. นพ. ยุทธพงศ์ วงศ์สวัสดิวัฒน์)

วันที่ ….…/………..............…/....….........

**18. ความเห็นจากหัวหน้าภาควิชา / หัวหน้าหน่วยงานต้นสังกัด / ประธานหลักสูตร (กรณีที่เป็นวิทยานิพนธ์ระดับบัณฑิตศึกษา)**

*(กรุณากาเครื่องหมายหน้าข้อ หรือให้ความเห็น พร้อมลงนามและระบุวันที่ เพื่อยืนยันการรับรองข้อเสนอโครงการวิจัย)*

........... หน่วยงาน/หลักสูตรอนุมัติและให้การสนับสนุนในการดำเนินการตามข้อเสนอโครงการวิจัยนี้

........... ผู้วิจัยหลักร่วมกับคณะวิจัยเป็นผู้มีความรู้ความสามารถ มีศักยภาพ และมีความพร้อมที่จะดำเนินงานวิจัยให้สำเร็จ โดยให้ความเคารพในศักดิ์ศรี สิทธิ และคำนึงถึงสวัสดิภาพของผู้เข้าร่วมวิจัยเป็นสำคัญ

........... ความเห็นอื่นๆ (ถ้ามี)

ลงชื่อ......................................................หัวหน้าภาควิชา/หน่วยงาน หรือประธานหลักสูตร

(.....................................................)

วันที่ ….…/………..............…/....….........
